# Advances in Fungal Phenaloenones—Natural Metabolites with Great Promise: Biosynthesis, Bioactivities, and an In Silico Evaluation of Their Potential as Human Glucose Transporter 1 Inhibitors

**DOI:** 10.3390/molecules27206797

**Published:** 2022-10-11

**Authors:** Sabrin R. M. Ibrahim, Abdelsattar M. Omar, Yosra A. Muhammad, Ali A. Alqarni, Abdullah M. Alshehri, Shaimaa G. A. Mohamed, Hossam M. Abdallah, Mahmoud A. Elfaky, Gamal A. Mohamed, Jianbo Xiao

**Affiliations:** 1 Department of Chemistry, Preparatory Year Program, Batterjee Medical College, Jeddah 21442, Saudi Arabia; 2 Department of Pharmacognosy, Faculty of Pharmacy, Assiut University, Assiut 71526, Egypt; 3 Department of Pharmaceutical Chemistry, Faculty of Pharmacy, King Abdulaziz University, Jeddah 21589, Saudi Arabia; 4 Center for Artificial Intelligence in Precision Medicines, King Abdulaziz University, Jeddah 21589, Saudi Arabia; 5 Department of Pharmaceutical Chemistry, Faculty of Pharmacy, Al-Azhar University, Cairo 11884, Egypt; 6 Pharmaceutical Care Department, Ministry of National Guard—Health Affairs, Jeddah 22384, Saudi Arabia; 7 Department of Natural Products and Alternative Medicine, Faculty of Pharmacy, King Abdulaziz University, Jeddah 21589, Saudi Arabia; 8 Faculty of Dentistry, British University, El Sherouk City, Suez Desert Road, Cairo 11837, Egypt; 9 Department of Pharmacognosy, Faculty of Pharmacy, Cairo University, Cairo 11562, Egypt; 10 Nutrition and Bromatology Group, Department of Analytical and Food Chemistry, Faculty of Sciences, Universidade de Vigo, 32004 Ourense, Spain

**Keywords:** phenaloenones, fungi, bioactivities, biosynthesis, human glucose transporter 1 (hGLUT1) inhibitors, in silico screening, molecular docking, molecular dynamics

## Abstract

Phenaloenones are structurally unique aromatic polyketides that have been reported in both microbial and plant sources. They possess a hydroxy perinaphthenone three-fused-ring system and exhibit diverse bioactivities, such as cytotoxic, antimicrobial, antioxidant, and anti-HIV properties, and tyrosinase, α-glucosidase, lipase, AchE (acetylcholinesterase), indoleamine 2,3-dioxygenase 1, angiotensin-I-converting enzyme, and tyrosine phosphatase inhibition. Moreover, they have a rich nucleophilic nucleus that has inspired many chemists and biologists to synthesize more of these related derivatives. The current review provides an overview of the reported phenalenones with a fungal origin, including their structures, sources, biosynthesis, and bioactivities. Moreover, more than 135 metabolites have been listed, and 71 references have been cited. SuperPred, an artificial intelligence (AI) webserver, was used to predict the potential targets for selected phenalenones. Among these targets, we chose human glucose transporter 1 (hGLUT1) for an extensive in silico study, as it shows high probability and model accuracy. Among them, aspergillussanones C (60) and G (60) possessed the highest negative docking scores of −15.082 and −14.829 kcal/mol, respectively, compared to the native inhibitor of 5RE (score: −11.206 kcal/mol). The MD (molecular dynamics) simulation revealed their stability in complexes with GLUT1 at 100 ns. The virtual screening study results open up a new therapeutic approach by using some phenalenones as hGLUT1 inhibitors, which might be a potential target for cancer therapy.

## 1. Introduction

**Scheme 1:**
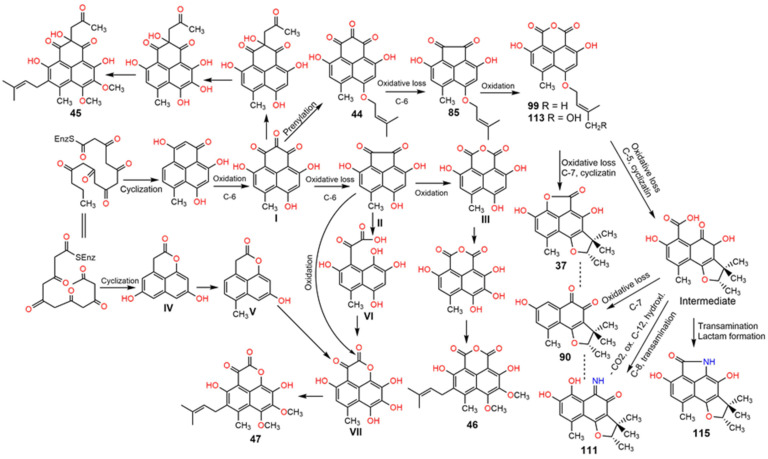
Biosynthetic pathway of compounds **37**, **44**–**47**, **85**, **90**, **99**, **111**, **113**, and **115** [20,31].

**Scheme 2:**
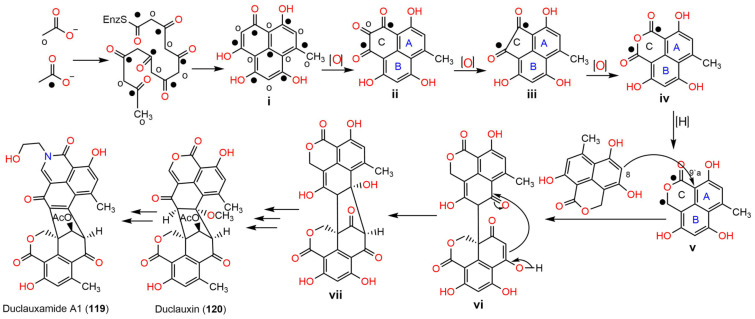
Biosynthetic pathway of compounds **119** and **120** [21,56,62,63].

**Scheme 3:**
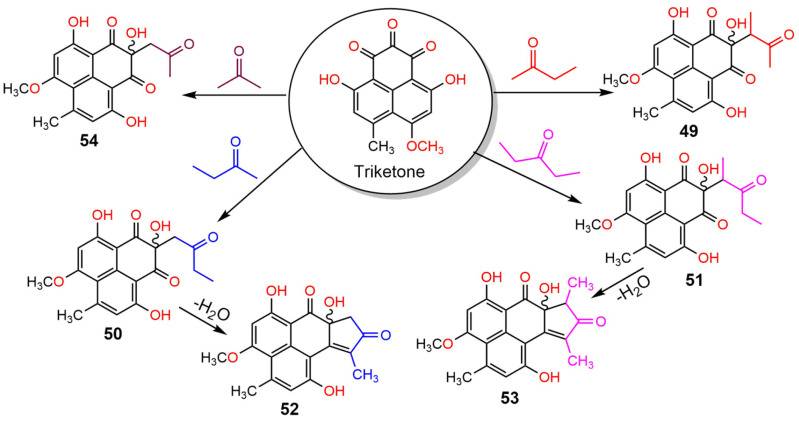
Proposed pathways for the formation of compounds **49**–**54** [48].

**Scheme 4:**
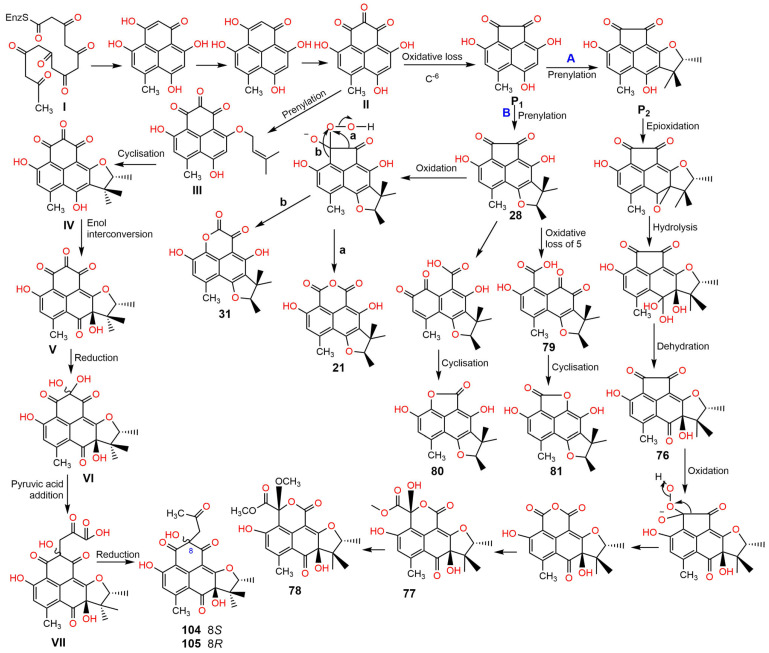
Biosynthetic pathway of compounds **21**, **28**, **31**, and **76–81** [21,39,46].

**Scheme 5:**
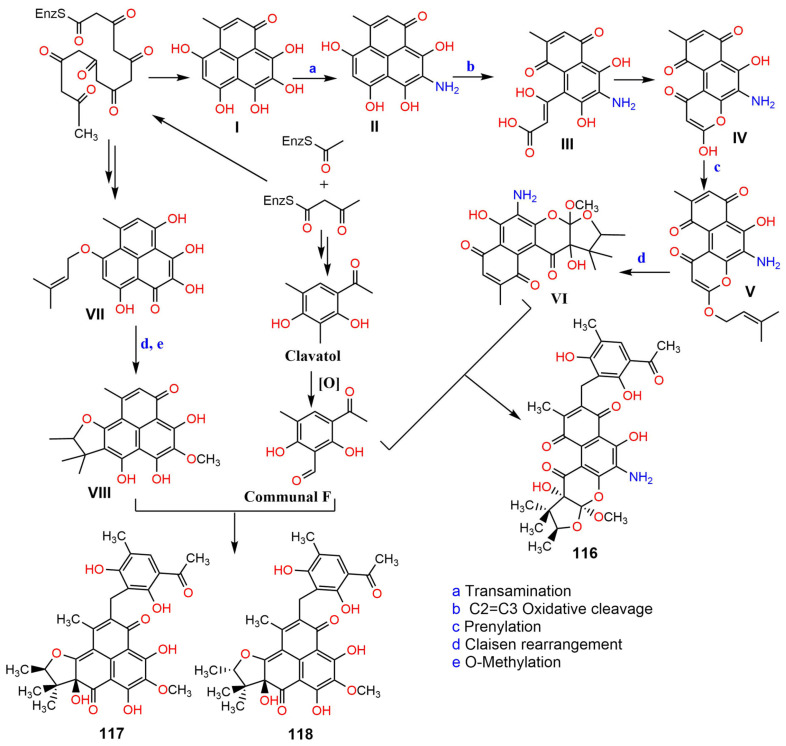
Biosynthetic pathway of compounds **116–118** [55,66].

**Figure 1:**
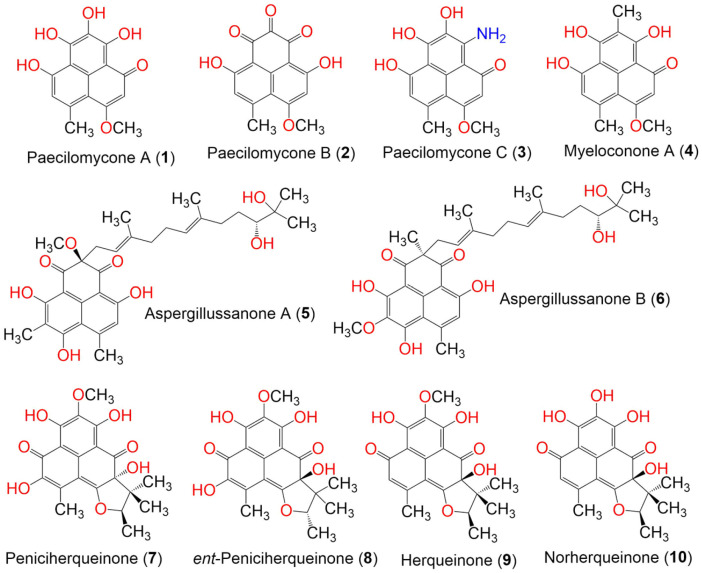
The structures of compounds **1–10**.

**Figure 2:**
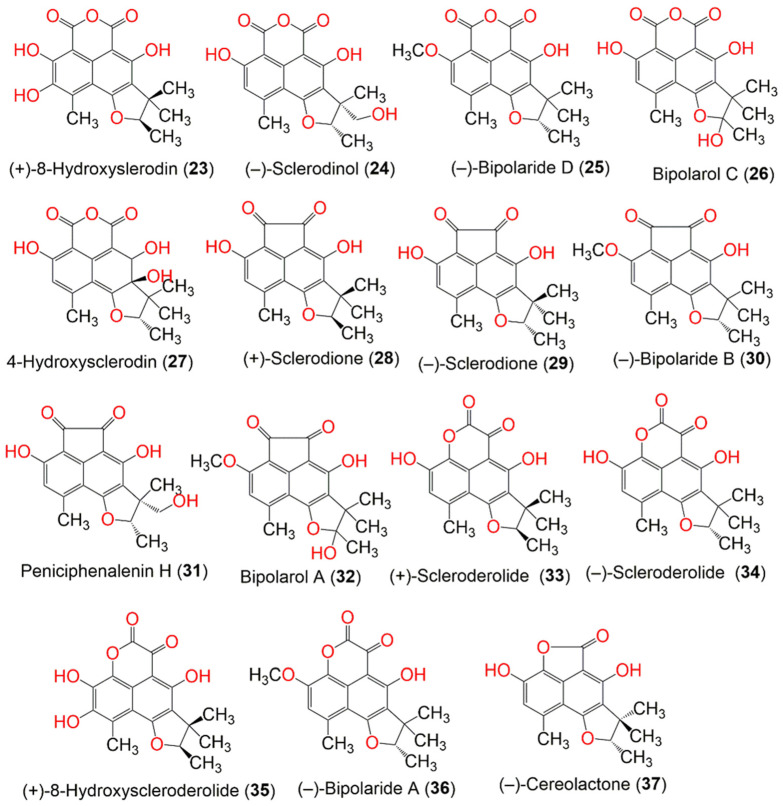
The structures of compounds **11–22**.

**Figure 3:**
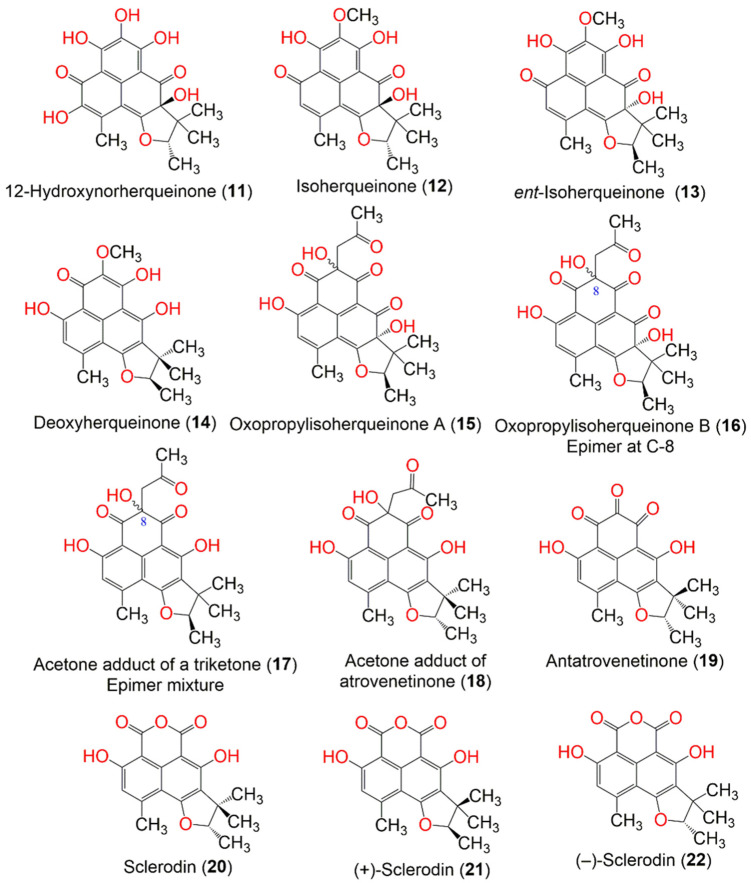
The structures of compounds **23–37**.

**Figure 4:**
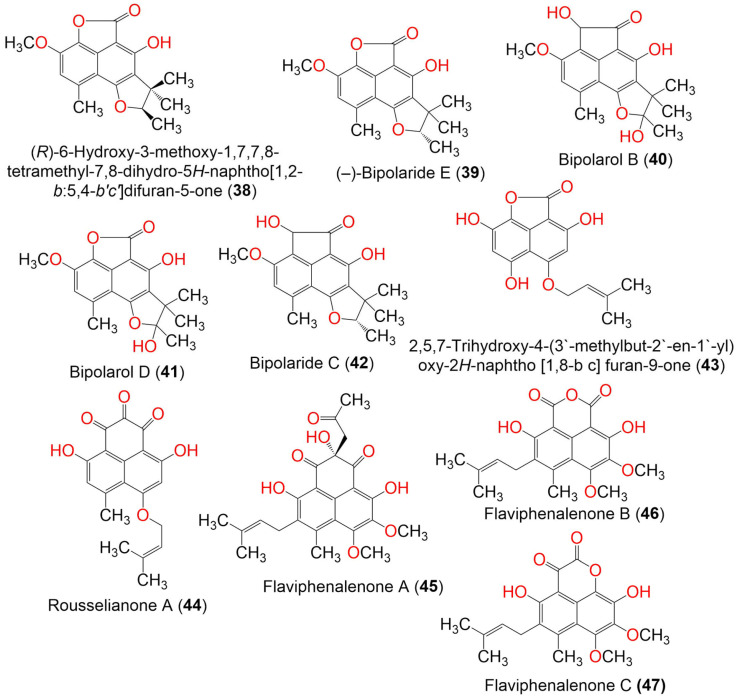
The structures of compounds **38**–**47**.

**Figure 5:**
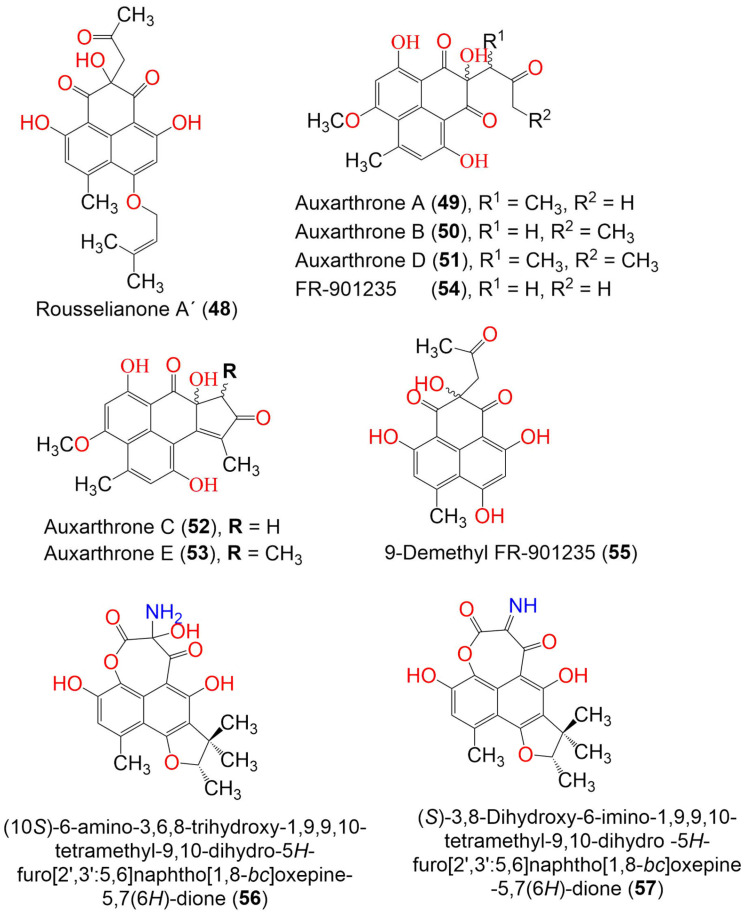
The structures of compounds **48–57**.

**Figure 6:**
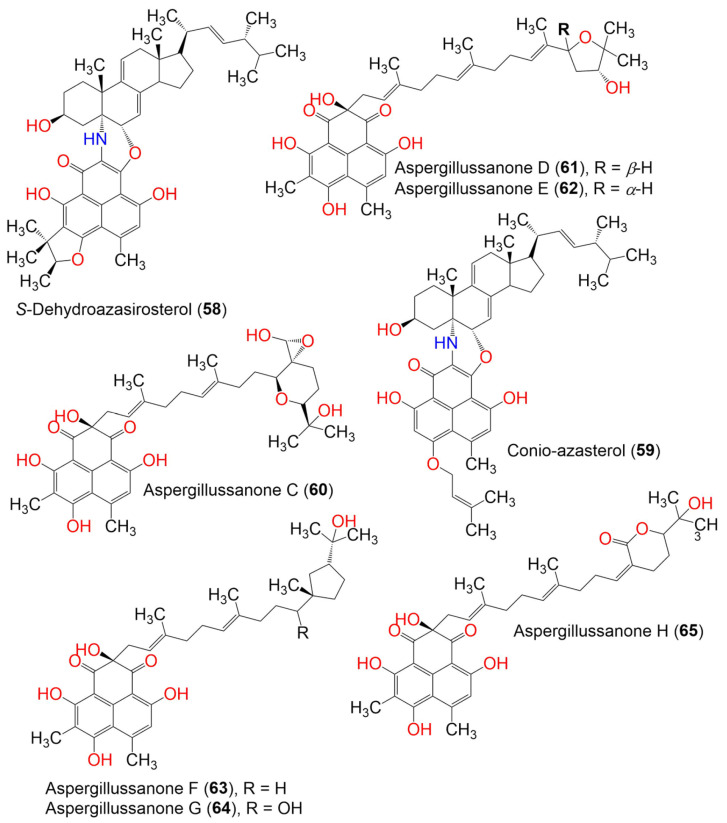
The structures of compounds **58–65**.

**Figure 7:**
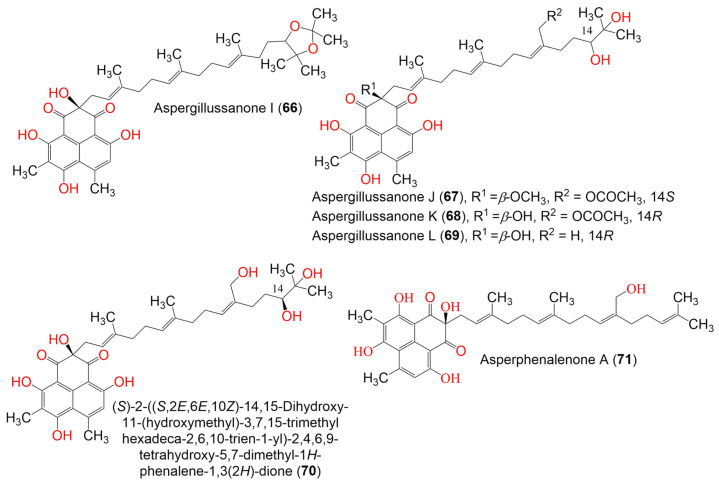
The structures of compounds **66–71**.

**Figure 8:**
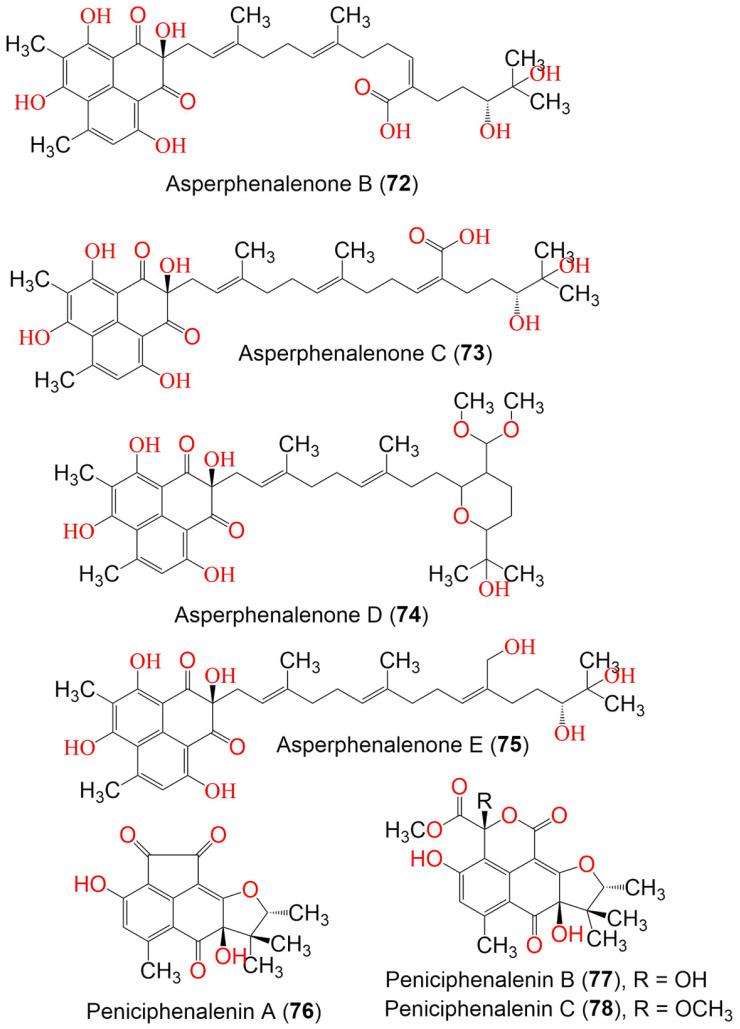
The structures of compounds **72**–**78**.

**Figure 9:**
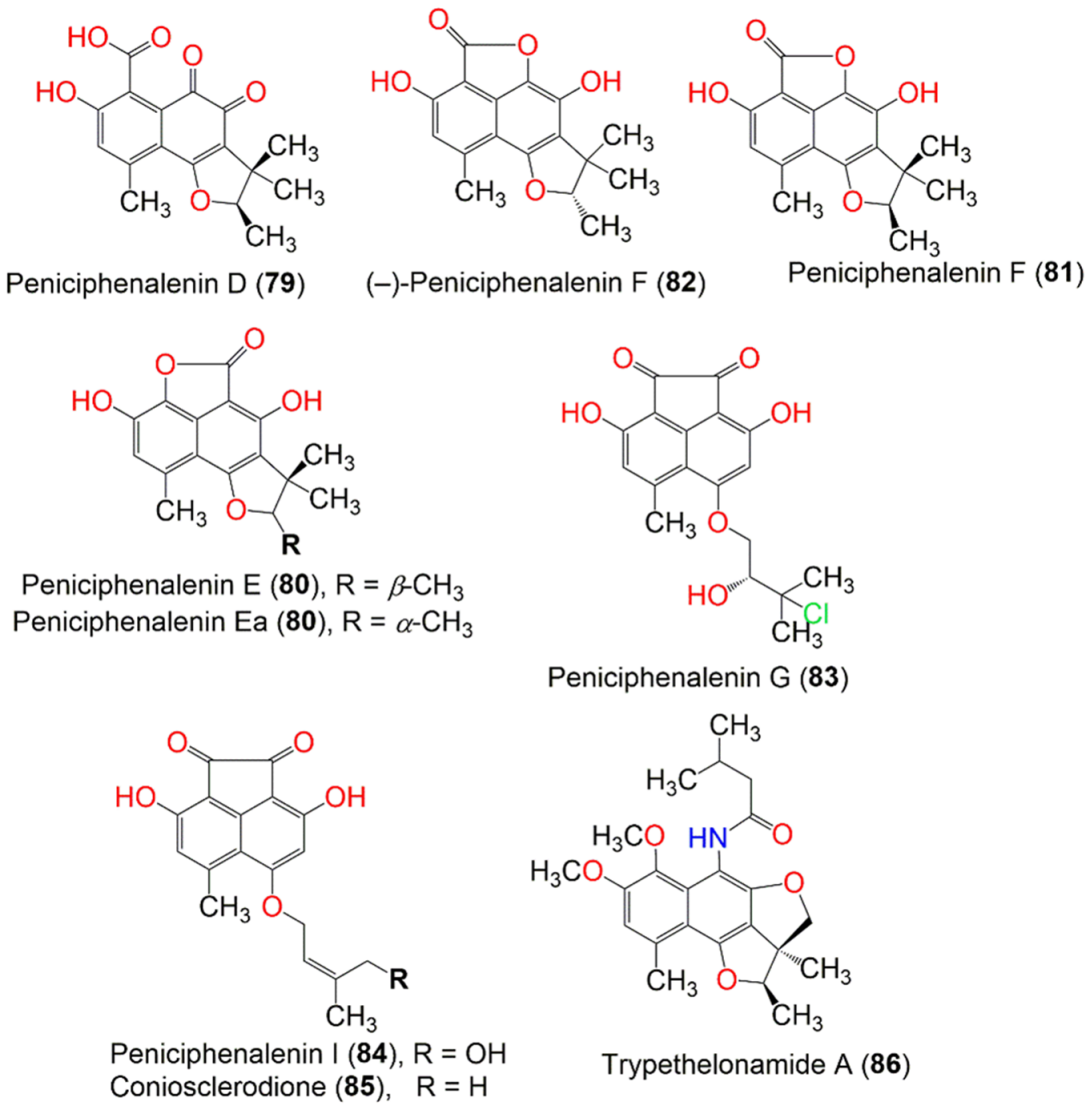
The structures of compounds **73**–**86**.

**Figure 10:**
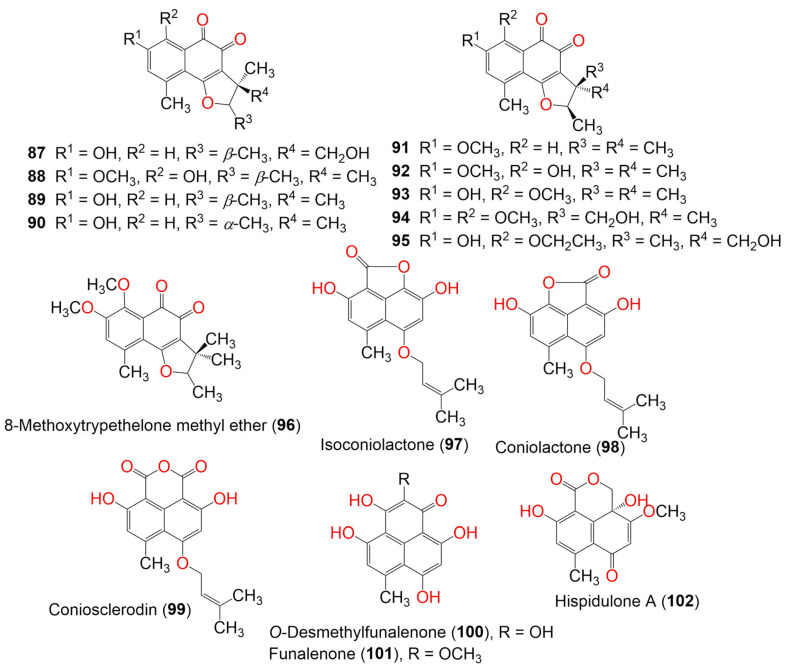
The structures of compounds **87**–**102**.

**Figure 11:**
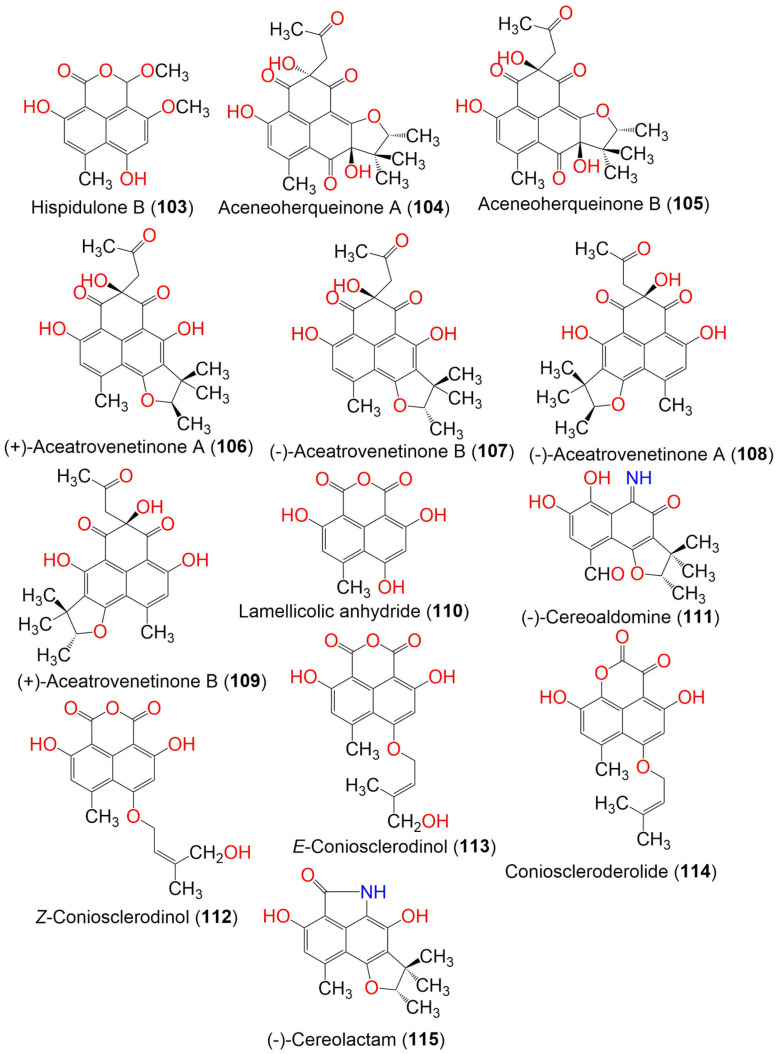
The structures of compounds **103**–**115**.

**Figure 12:**
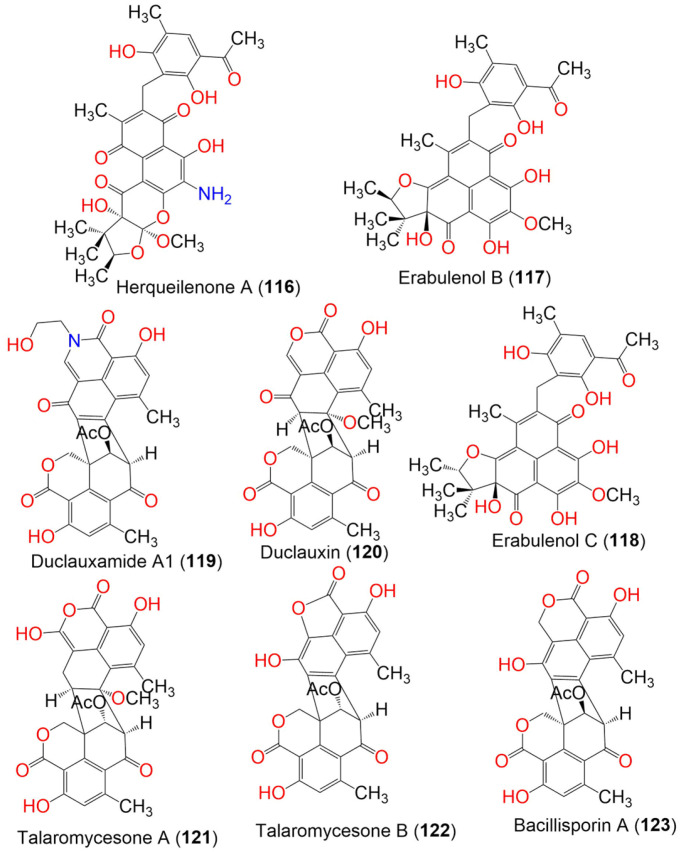
Structures of compounds **116**–**123**.

**Figure 13:**
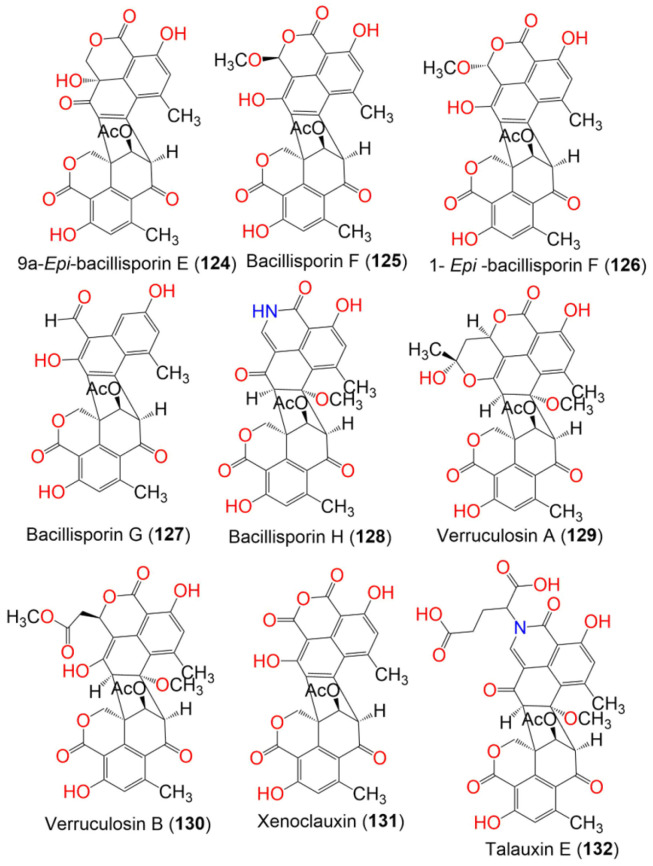
The structures of compounds **124**–**132**.

**Figure 14:**
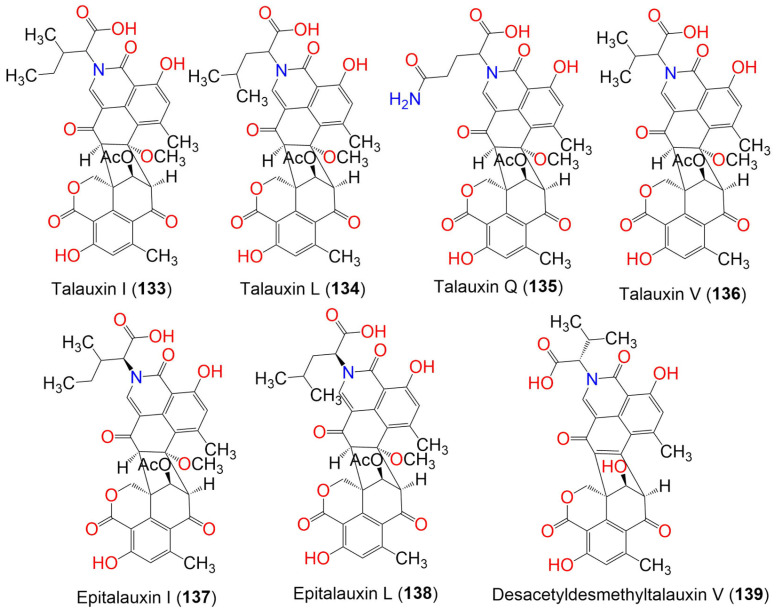
The structures of compounds **133**–**139**.

**Figure 15:**
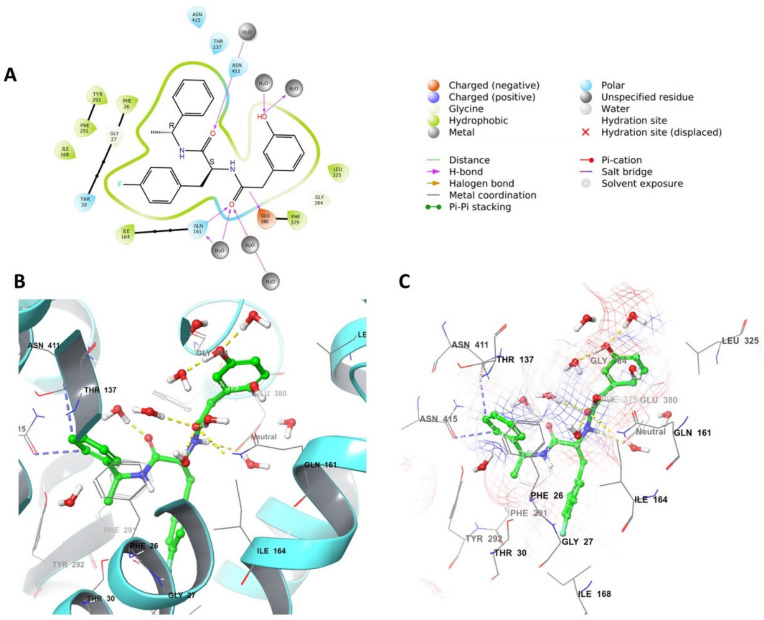
Docking validation by the re-docking of the native inhibitor into GLUT1 (PDB ID: 5EQG). (**A**) The interaction between the crystal structure 5EQG and the reference inhibitor **5RE** in 2D view. (**B**) The binding interactions in 3D view, and (**C**) 3D interaction with mesh surface view.

In the last few decades, fungi have attracted tremendous scientific attention due to their capability to biosynthesize diverse classes of bio-metabolites, with varied bioactivities that are utilized for pharmaceutical, medicinal, and agricultural applications [1,2,3,4,5,6,7,8,9,10,11,12,13,14]. Obviously, the number of reported biometabolites from a fungal origin is rapidly growing [15,16,17,18,19]. Fungi can produce a wide variety of structurally unique polyketide-derived metabolites; among them are phenalenones, in which various post-modifications, including prenylation, transamination, rearrangement, and oxidation diversify their structures [20,21,22]. Phenalenones belong to the aromatic ketones, consisting of a hydroxyl-perinaphthenone three-fused-ring system that has been reported as from both microbial and plant sources [21]. They are recognized as the higher plants’ phytoalexins, which confer resistance toward pathogens [23,24]. Phenalenones are also known as pollutants, resulting from the combustion of fossil fuels [21]. The first report of the isolation of a phenalenone derivative from a fungal source was in 1955 [25,26]. Fungal phenalenones have immense structural diversity, such as hetero- and homo-dimerization, and high degrees of nitrogenation and oxygenation, as well as the capacity to be complexed with metals, incorporating additional carbon frameworks or an isoprene unit by the formation of either a linear ether or a trimethyl-hydrofuran moiety [20,21]. Moreover, many acetone adducts of phenalenones were also reported that have an extended carbon chain at ring A, such as the acyclic diterpenoid adducts. These fungal metabolites have been demonstrated to exhibit a wide range of bioactivities, such as cytotoxic, antimicrobial, antioxidant, and anti-HIV, and tyrosinase, α-glucosidase, lipase, AchE (acetylcholinesterase), indoleamine 2,3-dioxygenase 1, angiotensin-I-converting enzyme, and tyrosine phosphatase inhibition. They are of great interest as potential lead compounds for synthetic organic chemistry because of the stability of their anions, phenalenyl radicals, and cations, as well as their interesting photophysical properties [27,28,29]. In a previous review, Elsebai et al. reported that up to the end of 2013, 72 phenalenone derivatives of fungal origin have been separated [21]. Phenalenones represent a rapidly growing class of bio-metabolites; therefore, an updated review is needed. In the current review, the fungal phenalenone derivatives that have been published from 2014 to 2021 have been summarized. Herein, 139 phenalenones have been listed along with their sources, bioactivities, and biosynthetic pathways (Tables 1 and 2, Schemes 1–5, and Figures 1–15). A literature search of the published studies was conducted over different databases: PubMed, Web of Science, Google Scholar, SciFinder, and Scopus, as well as through various publishers: SpringerLink, Wiley, Bentham, Taylor & Francis, Thieme Medical, and ACS.

The reported data regarding phenalenone derivatives revealed its anticancer potential; therefore, we chose human glucose transporter 1 (hGLUT1) for an extensive in silico study and, by using SuperPred “AI tools for targets prediction” and other computational tools, such as QikProp “ADMET characteristics prediction”, molecular docking to “measure the binding affinity between the ligands and the targets”, as well as MD (molecular dynamics) to assess the stability of the target–ligand interaction under simulated physiological circumstances, has been implemented.

## 2. Biosynthesis of Phenalenones

The phenalene nucleus is the basic structure of phenalenones, which are oxidized for a series of phenalenone derivatives. It was reported that fungal phenalenones (Table 1) are of polyketide origin, i.e., derived merely from acetate and malonate units [20,21,25,30]. Zhang et al. postulated the biosynthetic pathway of flaviphenalenones A–C (**45–47**) [31]. The heptaketide chain is first cyclized to produce a tricyclic aromatic skeleton, followed by oxidation and acetone addition at C-6, to form intermediate **I**. The subsequent addition of OH at the C-1, CH_3_ group and at C-14 and C-1, and of prenyl at C-10, yielded **45**. The oxidative loss of C-6 of **I** formed another intermediate **II** that was oxidized to yield the cyclic anhydride C (**III**) [20,21]. Successive C-1-hydroxylation, C-14/C-1 methylation, and the C-10 prenylation of **III** produced **46**. The cyclization of the heptaketide chain yielded another intermediate **IV** that underwent dehydroxylation at C-10 and C-11 methylation, to generate **V**. Lastly, the methylation, oxidation, and prenylation of **V** gave **47**. The immediate oxidation or oxidative cleavage of **II** and the subsequent lactonization of **VI** produced **VII**, which was methylated and prenylated to yield **47** [31] (Scheme 1). Moreover, Nazir et al. utilized [1–^13^C]-labeled acetate to postulate the biosynthetic pathways for **37**, **44**, **85**, **90**, **98**, **111**, **113**, and **115**, which is biosynthesized by the marine algae-derived *Coniothyrium cereale*. Nazir et al. hypothesized that these metabolites originated from a common joint heptaketide precursor that underwent successive oxidative cleavage reactions [20]. Some of them (e.g., **90**, **98**, **111**, and **115**) are hexaketides with methyl groups that are added through methyltransferases (Scheme 1). Moreover, it was suggested that the polyketide skeleton of **115** was formed through the degradation of heptaketide by the loss of two carbon atoms [20].

Duclauxin (**120**) is an oligophenalenone heptacyclic dimer, consisting of dihydroisocoumarin and isocoumarin units that are joined together by a cyclopentane ring. The previously reported labeling experiment revealed that **120** originated from a heptaketide chain, which was cyclized to produce phenalenone (**i**) [21,61]. A triketone (**ii**) was formed by the oxidative loss of one of its carbons to yield a contracted ring, C. Its decarboxylation and regio-selective oxygen insertion, induced by enzymes or air, yielded dione (**iii**) and naphthalic anhydride (**iv**) [21]. Then, the selective reduction of (**iv**) generated a lactone (**v**). The dimerization of two lactone units through oxidative radical coupling between C-8 and C-9′a, which was catalyzed by oxidative enzymes, yielded a biaryl (**vi**) [62,63]. The latter underwent an intramolecular aldol condensation between the C-8′ and C-7 ketone group to furnish the aldol fragment (**vii**). The latter could experience a group of successive tailoring modifications in terms of reduction, methylation, acetylation, and dehydration, to produce **120** [56,64]. Compound **119** was assumed to be biosynthesized through ammonolysis, with the aid of one serine as a nitrogen donor and a further serine moiety′s decarboxylation, to provide **119** (Scheme 2) [56].

On the other hand, it was hypothesized that the compounds **49**, **50**, **52**, and **54** were artifacts, resulting from the spontaneous addition of acetone or methyl ethyl ketone to the unstable triketone (Scheme 3) [48]. Meanwhile, the use of 3-pentanone as an initial extracting solvent ultimately led to the formation of **51** and **53** [48].

Li et al. proposed a biosynthetic pathway for compounds **21**, **28**, **31**, and **76**–**81**, which have a phenalenone nucleus fused to a trimethylfuran ring [39]. The trimethylfuran ring was biologically related to mevalonic acid [65]. The oxidative loss of C-6 of the heptaketide-derived phenalenone nucleus yielded **P1**, which could be prenylated in two various paths (**A** and **B**), leading to the formation of **P2** and **28**. The enzymatic epoxidation of the **P2** double bond, followed by hydrolysis and then dehydration, yielded **76**. The later oxidation produced **77** and **78**. Similarly, the oxidation of **28** resulted in the formation of **21** and **31**. Compound **28** was a precursor of **79**–**81** by the oxidative loss of C-7 (**80**) or C-5 (**79** and **81**) and then formed a lactone ring (**80** and **81**) [39]. The linkage of a prenyl side chain to 5-OH of the tricyclic intermediate **II** was catalyzed by prenyltransferase to yield the prenylated intermediate (**III**). Additionally, **IV** was generated from the Claisen rearrangement and cyclization of **III** [21] (Scheme 4).

The enol inter-conversion of the C-4 and C-3 of **IV** generated an intermediate **V** that was converted by reduction to form **VI**, which was very active due to the C-8 two hydroxyl groups. Then, pyruvic acid was added to one of the hydroxyl groups to give **VII**. Finally, **104** and **105** were produced by the **VII** COOH group reduction, which was catalyzed by reductase [46]. Yu et al. proposed biosynthetic pathways for **116–118** (Scheme 5). The intermediate **I** was trans-aminated to yield **II**, which underwent oxidative cleavage to form a naphthoquinone (**III**) [55]. Then, a benzo[*f*]chromene-1,7,10-trione derivative (**IV**) was generated through the cyclization between 7-OH and the 3-carbonyl group. The prenylation of **IV**, followed by a Claisen rearrangement, yielded **VI**. The coupling of communal F with **VI** generated **116** [66]. Furthermore, **112** and **117** were produced from the coupling between communal F and **VIII** [55].

## 3. Bioactivities of Phenalenones

The bioactivities of some of the reported metabolites have been investigated. In this regard, 70 metabolites have been associated with some type of biological action, including cytotoxic, antimalarial, antimycobacterial, anti-inflammatory, anti-angiogenic, immunosuppressive, and antioxidant properties, as well as IDO1, α-glucosidase (AG), ACE, tyrosinase, and PTP inhibition. This information has been discussed and listed in Table 2.

Paecilomycones A–C (**1**–**3**) were purified from *Paecilomyces gunnii* culture extract with the aid of a preparatory HSCCC, guided by HPLC-HRESIMS, used as a tyrosinase inhibitor. Compound **1** was similar to myeloconone A2 (**4**), which was formerly separated from the lichen *Myeloconis erumpens* [67], except that **1** has an OH group at C-8 instead of an OCH_3_ group. Compound **3** was deduced as 9-amino-6,7,8-trihydroxy-3-methoxy-4-methyl-1H-phenalen-1-one; the existence of NH_2_ in **3** was confirmed by a positive purple reaction with a ninhydrin reagent in the TLC plate. They were characterized by means of spectroscopic analyses. These metabolites exhibited potent tyrosinase inhibitory potential (IC_50_s 0.11, 0.17, and 0.14 mM, respectively) in the form of kojic acid (IC_50_ 0.10 mM), being stronger than arbutin (IC_50_ 0.20 mM). This influence was found to be positively related to the number of OH groups [32] (Figure 1).

Aspergillussanones A (**5**) and B (**6**) were separated from *Aspergillus* sp. PSU-RSPG185 broth extract. They differed from each other in the substitutions at C-8 and C-4, as well as in the C-4 configuration. The configuration of their double bonds was determined to be *E,* based on signal enhancement in the NOEDIFF experiment, and the 4*S* and 10′*R* in **5** and 4*R* and 10′*R* in **6** was assigned by the CD spectrum. Only compound **5** exhibited weak cytotoxic activity toward Vero cells and KB (IC_50_s 34.2 and 48.4 μM, respectively) in the resazurin microplate assay, compared to ellipticine (IC_50_ 4.5 and 4.1 μM, respectively), whereas **6** was inactive against the tested cell lines. Additionally, they showed no antimalarial or antimycobacterial potential toward *Plasmodium falciparum* and *Mycobacterium tuberculosis* when using GFP (green fluorescent protein) and the micro-culture radioisotope technique, respectively [33] (Figure 1).

Eleven metabolites of the herqueinone subclass, including six new derivatives, *ent*-peniciherqueinone (**8**), 12-hydroxynorherqueinone (**11**), *ent*-isoherqueinone (**13**), oxopropylisoherqueinones A (**15**) and B (**16**), and 4-hydroxysclerodin (**27**) and the known analogs, **9**, **12**, **17**, **22**, and **34** were extracted from a marine-derived *Penicillium* sp. (Figure 2). The new metabolites’ configuration was assigned, based on specific rotations and chemical modifications. Compound **17** exhibited moderate anti-inflammatory activity (IC_50_ 3.2 µM) towards mouse macrophage RAW 264.7 cells, compared to AMT (IC_50_ 0.2 µM) in the nitric oxide synthase assay. In addition, **27** exhibited moderate anti-angiogenic potential (IC_50_ 20.9 µM) toward HUVECs (human umbilical vascular endothelial cells), compared to sunitinib (IC_50_ 1.5 µM), in the tube formation assay. Furthermore, **8** and **12** moderately induced adipogenesis (IC_50_ 57.5 and 39.7 µM, respectively) in the hBM-MSCs (human bone marrow-mesenchymal stem cells), compared to pioglitazone (IC_50_ 0.69 µM), in the adiponectin production assay [35].

Lee et al. purified **18** from a culture of *Penicillium herquei* FT729, derived from Hawaiian volcanic soil by LC-MS-guided chemical analysis. It was identified by spectroscopic analysis, optical rotation, and LC-MS analysis. The pretreatment of T cells with **18** remarkably reduced IL-2 production and the expression of surface molecules, including CD-25 and -69, and activated T cell proliferation after TCR-mediated stimulation, as well as abrogating the NF-κB and MAPK pathways. Therefore, it effectively down-regulated T cell activity via the MAPK pathway, which indicated its immunosuppressive potential [38]. Furthermore, *P. herquei* PSURSPG93, obtained from soil, produced a new derivative, peniciherqueinone (**7**), along with the formerly separated derivatives: herqueinone (**9**), deoxyherqueinone (**14**), the acetone adduct of atrovenetinone (**18**) (as a mixture of epimers), sclerodin (**20**), and (−)-7,8-dihydro-3,6-dihydroxy-1,7,7,8-tetramethyl-5H-furo-[2′,3′,:5,6]naphtho[1,8-*bc*]furan-5-one (**37**). Compound **7** was structurally similar to **9**, except for the disappearance of one olefinic proton signal. Its *R*-configuration at C-4 was determined by an anisotropic effect and CD spectroscopy, which was opposite to **9**. Compounds **9**, **14**, and **20** had no cytotoxic effect toward MCF-7, KB, and noncancerous Vero cell lines. In addition, only **9** exhibited mild antioxidant potential, where it inhibited OH^•^, DPPH^•^, and O_2_^•−^ (IC_50_ 0.48, 6.34, and 4.11 mM, respectively) in the hydroxyl radical, DPPH, and superoxide radical scavenging assays, respectively, in comparison with tannic acid (OH^•^, IC_50_ 0.26), butylated hydroxytoluene (DPPH^•^, IC_50_ 0.11), and trolox (O_2_^•−^, IC_50_ 0.96 mM) [34].

Intaraudom et al. purified the new derivatives, **25**, **26**, **30**, **32**, **36**, and **39–42**, together with **22** and **34**, from the broth EtOAc extract of the marine-derived *Lophiostoma bipolare* BCC25910 (Figure 3). Their structures were assigned via spectroscopic analysis, whereas the C-2′, S-configuration was determined based on X-ray analysis, a chemical reaction, and a specific optical rotation negative sign. They showed no antimalarial activity toward the *P. falciparum* K-1 strain and no antifungal activity toward *C. albicans*. On the other hand, **25**, **26**, **36**, **39**, and **40** showed moderate antibacterial potential toward *B. cereus* (MICs 12.5 µg/mL). However, other compounds were inactive against *B. cereus* (concentration 25 µg/mL). Additionally, they exhibited weak cytotoxicity toward KB, MCF-7, NCI-H187, and Vero cells [42].

Macabeo et al. purified **29**, **34**, and **90** from a culture of *Pseudolophiostoma* sp. MFLUCC-17-2081 obtained from a dried branch of *Clematis fulvicoma*. Compounds **29** and **34** conferred more potent α-glucosidase inhibition (IC_50_ 48.7 and 120 µM, respectively) than N-deoxynojirimycin (IC_50_ 130.5 µM). They also potently inhibited the hydrolysis of *p*-nitro-phenylbutyrate, using porcine lipase. Interestingly, **29** and **34** showed stronger inhibitory potential (IC_50_s 1.0 and 3.4 µM, respectively) than orlistat (IC_50_ 9.4 µM). The in silico techniques employed revealed that **29** and **34** exhibited strong binding affinities to porcine pancreatic lipase and α-glucosidase through π–π and H-bonding interactions, while **90** was weakly active (IC_50_ > 100 µM) toward both enzymes [45].

Zhang et al. purified new derivatives, flaviphenalenones A–C (**45**–**47**), from solid cultures of *Aspergillus flavipes* PJ03-11 (Figure 4). The 6*S* absolute configuration of **45** was determined by the computational ECD method. Compound **47** was a positional isomer of **46**. They represented the first report of phenalenones with a directly connected C-10 isoprene unit, whereas **47** had a keto-lactone group at C-8. Compounds **46** and **47** possessed potent α-glucosidase inhibitory potential (IC_50_s 94.95 and 78.96 µM, respectively) than acarbose (IC_50_ 685.36 µM). On the other hand, **45** displayed significant cytotoxic capacities toward MCF-7 and A549 (IC_50_ 10.0 and 6.6 µg/mL, respectively) compared to doxorubicin (IC_50_ 0.4 and 0.2 µg/mL, respectively), while **47** showed moderate cytotoxicity toward A549 (IC_50_ 28.5 µg/mL) [31].

Auxarthrones A–E (**49**–**53**) and FR-901235 (**54**) were obtained from the culture of the coprophilous fungus *Auxarthron pseudauxarthron* TTI-0363 (Figure 5). Compounds **52** and **53** possessed an unusual 7a,8-dihydrocyclopenta[*a*]phenalene-7,9-dione ring system. Compound **49** was separated into a mixture of racemic diastereomers; their structures were confirmed by X-ray crystallography. Compounds **49** and **51** showed moderate antifungal potential toward *C. albicans* and *C. neoformans* (MICs 6.4 and 3.2 μg/mL, respectively), compared to amphotericin B (MIC 0.8 μg/mL). The other phenalenones were weakly active (MIC ranging from 6.4 to 51.2 μg/mL). On the other hand, they showed no significant cytotoxic effects against MDA-MB-451 and MDA-MB-231 [48].

Compound **56** obtained from the marine-derived endophytic fungus, *Coniothyrium cereale,* harboring the Baltic Sea algae *Enteromorpha* sp., which had unprecedented imine functionality between two carbonyls to produce an oxepane-imine-dione ring. It exhibited a moderate cytotoxic potential toward the SKM1, U266, and K562 cancer cell lines (IC_50_s 75.0, 45.0, and 8.5 µM, respectively) in the MTT assay [37]. The new phenalenone derivatives, aspergillussanones C–L (**60**–**69**), along with the known analog **70**, were isolated from the solid culture of *Aspergillus* sp. that was associated with *Pinellia ternate* (Figure 6 and Figure 7).

Compounds **60**–**69** are unusual acyclic diterpenoid adducts that are partly epoxidized and variously oxidized to produce diverse heterocyclic analogs. Their structures and absolute configurations were established by spectroscopic, ECD, and Mo_2_(OCOCH_3_)_4_-induced ECD analyses. Their antibacterial effectiveness toward *Pseudomonas* *aeruginosa,* *Escherichia coli,* *Staphylococcus aureus,* and *Bacillus subtilis* was evaluated using the broth micro-dilution method. Compound **69** exhibited the most potent antibacterial potential against *B. subtilis, S. aureus,* and *P. aeruginosa* (MIC 4.80, 2.77, and 1.87 μg/mL, respectively), compared to streptomycin (MIC 0.34 μg/mL for *P. aeruginosa*) and penicillin (MIC 0.063 and 0.13 μg/mL for *S. aureus* and *B. subtilis,* respectively). Compounds **65**–**67** had potential versus *P. aeruginosa* (MIC_50_s 6.55–12.00 μg/mL). Meanwhile, **62**, **63**, **65**, and **67** showed significant activity toward *E. coli* (MIC 3.93–7.83 μg/mL) [50].

*Aspergillus* sp. CPCC 400735, which is associated with *Kadsura longipedunculata,* was found to biosynthesize the structurally unusual phenalenones, asperphenalenones A–E (**71**–**75**), these having a linear diterpene moiety that is connected to the phenalenone skeleton through a C-C bond (Figure 8). Their structures were established from extensive NMR spectroscopic analyses, while the absolute configuration was determined based on the CD spectra. Compounds **71** and **74** exhibited anti-HIV activity (IC_50_ 4.5 and 2.4 µM, respectively), in comparison to lamivudine (IC_50_ 0.1 µM) and efavirenz (IC_50_ 0.0004 µM), using SupT1 cells in the luciferase assay system, while **72** and **75** exhibited weak activity (IC_50_ 32.6 and 22.1 µM, respectively) [51].

The new derivatives, peniciphenalenins A–F (**76**–**81**), along with the formerly reported **21**, **28**, and **33**, were obtained from *Penicillium* sp. ZZ901 culture, using ODS and HPLC (Figure 9). Their structures were determined by extensive spectroscopic analysis, ECD calculation, optical rotation, and single X-ray diffraction. The analyses identified a phenalenone skeleton, fused to a trimethyl-furan ring. Compounds **28** and **33** showed antimicrobial activity toward MRSA and *E. coli* (MICs 23–35 µg/mL for **28** and 7.0–9.0 µg/mL for **33**). On the other hand, **21**, **28**, and **33** showed weak antiproliferative activity against the glioma cells (IC_50_ 23.24–6.93 µM), compared to doxorubicin (IC_50_ 1.2 and 0.47 µM, respectively) [39].

Han et al. separated three new red-colored phenalenone derivatives, peniciphenalenins G–I (**83**, **31**, and **84**), along with coniosclerodione (**85**) and (−) sclerodinol (**24**) from the marine sediment-derived fungus, *Pleosporales* sp. HDN1811400, using UV-HPLC guided investigation. Their absolute configurations were determined by detailed spectroscopic and ECD analyses, in addition to the chemical method. Compound **83** was the first example of a chlorinated phenalenone derivative. Compounds **24, 31**, **83**, and **85** showed antimicrobial potential versus *B. cereus,* *Proteus* sp., *M. phlei,* *B. subtilis,* *V. parahemolyticus,* *E. tarda,* MRCNS, and MRSA (MICs 6.25–50.0 µM). Compound **85** (MIC 6.25 µM) was more active than compound **84**, indicating that 19-OH reduced the activity. Notably, compounds **24**, **31**, **83**, and **85** showed better inhibitory potential toward MRCNS and MRSA than that of ciprofloxacin, indicating their potential regarding drug-resistant strains [44].

Basnet et al. reported the isolation of a new yellow compound, trypethelonamide A (**86**), and a new dark violet-red compound, 5′,-hydroxytrypethelone (**87**), along with a dark violet-red metabolites (+)-8-hydroxy-7-methoxytrypethelone (**88**), (+)-trypethelone (**89**), and (−)-trypethelone (**90**) from the cultured lichenized fungus *Trypethelium eluteriae* by using Sephadex LH-20, ODS, SiO_2_, and HPLC. They were fully characterized via spectroscopic and ECD spectral analyses (Figure 10). They showed moderate to weak cytotoxicity versus the RKO cell line (IC_50_ ranged from 22.6 to 113.5 µM), compared to taxol (IC_50_ 0.05 µM) in the CCK8 assay, while they had no antioxidant potential in the DPPH assay (concentration 200 µM) [52].

Two new metabolites, 8-methoxytrypethelone (**93**) and 5′-hydroxy-8-ethoxytrypethelone (**95**), along with compounds **20**, **38**, **89**, **91**, **92**, and **94** were separated from mycobiont culture of *Trypethelium eluteriae* by preparative TLC and column chromatography. They were fully characterized by using spectroscopic, ECD, and X-ray analyses. Compound **89** (MIC 12.5 µg/mL) showed potent antimycobacterial potential toward *M. tuberculosis,* followed by **38** and **94** (MIC 50.0 µg/mL). Moreover, **89** had moderate potential (MIC 25.0 µg/mL) toward *M*. *chitae,* *M*. *szulgai, M*. *phlei,* *M*. *flavescens,* *M*. *parafortuitum,* and *M*. *kansasii*. In addition, compounds **89** and **94** were active versus *S. aureus* (MIC 25 µg/mL) [41]. Funalenone (**101**) was also purified as a PTP inhibitor from a marine-derived fungal strain of *Aspergillus* sp. SF-5929 and was tested for its inhibitory potential on *h*PTP1B_1-400_ in a photocolorimetric assay using the *h*PTP1B_1_ enzyme. It exhibited powerful PTP1B inhibitory potential (IC_50_ 6.1 µM), compared to ursolic acid (IC_50_ 4.3 µM). It was found that **101** was a noncompetitive PTP1B inhibitor that targeted the active or allosteric site of the enzyme [53]. *Chaetosphaeronema hispidulum* yielded two new phenalenones, hispidulones A (**102**) and B (**103**), which were assigned by spectroscopic and ECD analyses (Figure 11).

Compound **102** had a cyclohexa-2,5-dien-1-one moiety, whereas **103** possessed a hemiacetal OCH_3_ group that was uncommon in phenalenone analogs. Compound **103** showed cytotoxic potential toward A-549, Huh7, and HeLa cells (IC_50_ 2.71, 22.93, and 23.94 μM, respectively), compared with *cis*-platinum (IC_50_ 8.73, 5.89, and 14.68 μM, respectively), whereas **102** did not show any effect in the MTT assay [54].

Aceneoherqueinones A (**104**) and B (**105**), (+)-aceatrovenetinone A (**106**), and (+)-aceatrovenetinone B (**109**), along with the known congeners, (+)-scleroderolide (**33**), (−)-scleroderolide (**34**), (−)-aceatrovenetinone B (**107**), and (−)-aceatrovenetinone A (**108**), were reported from the marine mangrove-derived fungus, *Penicillium herquei* MA-370. Among these, compounds **104** and **105** were rare phenalenones, having a cyclic ether unit between C-2′ and C-5 (Figure 11). Compounds **106**–**109** were unstable stereoisomers, possessing configurationally labile chiral centers that were characterized by HPLC-ECD analyses, assisted by TDDFT-ECD calculations. The absolute configuration of **104** was confirmed by X-ray, while those of **105**–**109** were established by ECD spectra TDDFT-ECD calculations. Compounds **104** and **105** displayed ACE (angiotensin-I-converting enzyme) inhibitory activity (IC_50_s 3.10 and 11.28 μM, respectively), compared to captopril (IC_50_ 9.23 nM). The molecular docking study revealed that compound **104** bound well with ACE via hydrogen interactions with the residues Gln618, Ala261, Asn624, and Trp621, while **105** interacted with the Tyr360 and Asp358 residues. This difference in interactions was likely caused by the C-8 epimerization of both compounds [46].

*Penicillium herquei* FT729, which is associated with Hawaiian volcanic soil, yielded herqueilenone A (**116**) and erabulenols B (**117**) and C (**118**) (Figure 12). Their structures were determined by spectroscopic analysis, ECD calculations, and GIAO (gauge-including atomic orbital) NMR chemical shifts. Compounds **117** and **118** exhibited significant IDO1 (indoleamine 2,3-dioxygenase 1) inhibitory activities (with IC_50_ values of 13.69 and 14.38 µM, respectively), compared to epacadostat (IC_50_ 0.015 µM). Therefore, they can be developed into cancer immunotherapeutics. Compounds **117** and **118** also exhibited a protective effect toward acetaldehyde-induced damage in PC-12 cells and significantly increased cell viability [55].

Duclauxamide A1 (**119**), a new polyketide heptacyclic-oligophenalenone dimer with an *N*-2-hydroxyethyl moiety, was isolated from *Penicillium manginii* YIM PH30375, which is associated with *Panax notoginseng*. It belongs to the 9′S-duclauxin epimers, based on spectroscopic data analysis, single-crystal X-ray diffraction, and the computational ^13^C NMR-DFT method. It is structurally related to duclauxin (**120**), showing the replacement of the *O*-atom with the *N*-containing chain, without modification, on the original carbon skeleton. It showed moderate cytotoxicity toward MCF-7, SMML-7721, A-549, HL-60, and SW480 (IC_50_ ranged from 11 to 32 μM), compared to cisplatin and paclitaxel [56]. Two new oxaphenalenone dimers, talaromycesones A (**121**) and B (**122**), were isolated from the marine fungus *Talaromyces* sp. LF458 culture broth and mycelia. Their relative configuration was determined by NOESY spectral data. Compound **116** was the first metabolite with a 1-nor oxaphenalenone dimer framework. They exhibited significant antibacterial potential toward *S*. *epidermidis* and *S. aureus* (IC_50_s 3.70 and 5.48 μM, respectively, for **121**, and 17.36 and 19.50, respectively, for **122**), compared to chloramphenicol (IC_50_ 1.81 and 2.46 μM, respectively) in the resazurin microplate assay. They revealed no antifungal effectiveness toward *Trichophyton rubrum* and *C. albicans*. Moreover, **121** exhibited AchE (acetylcholinesterase) inhibition (IC_50_ 7.49 μM) that was more powerful than huperzine (IC_50_, 11.60 μM) in the modified Ellman’s enzyme/immunosorbent assay [59].

In the case of 9a-*epi*-bacillisporin E (**124**) and bacillisporins F–H (**125**, **127**, and **128**), new oligophenalenone dimers, along with bacillisporin A (**123**), were separated from a culture of *Talaromyces stipitatus* (Figure 13). Their absolute configurations and structures were determined based on spectroscopic analyses, ECD, and GIAO NMR shift calculation, followed by DP4 probability analysis. Only **128** was moderately active (IC_50_ 49.5 µM) toward the HeLa cell, compared to cisplatin (IC_50_ 10.6 µM). No effect was observed on the growth of *E. coli* (IC_50_ > 100 μg/mL) for all isolated compounds, while **123** displayed noticeable antibacterial potential versus *Staphylococcus hemolyticus,* *S*. *aureus* (ATCC 6538), and *Enterococcus faecalis* (MICs 9.5, 5.2, and 2.4 μg/mL, respectively), compared to tetracycline (MICs 29.2, 0.05, and 0.4 μg/mL, respectively). However, **128** had an observable effect on *S. aureus* (MIC 5.0 μg/mL) when using a microtiter plate assay [60].

*Talaromyces verruculosus* yielded two new oligophenalenone dimers, verruculosins A (**129**) and B (**130**), and the related known analogs, duclauxin (**120**), bacillisporin F (**125**), and xenoclauxin (**131**) (Figure 13). Compound **129** was a novel oligophenalenone dimer with a unique octacyclic skeleton. Compounds **129** and **130** were fully characterized by spectroscopic, X-ray crystallography, and ECD analyses as well as, optical rotation and NMR calculations. Compounds **120**, **125**, **129**, and **131** exhibited potent CDC25B inhibitory activities (IC_50_ values of 0.75, 0.40, 0.38, and 0.26 µM, respectively), compared to Na_3_VO_4_ (IC_50_ 0.52 µM). In addition, **120** and **129**–**131** displayed moderate EGFRIC inhibitory activities (IC_50_ values from 0.24 to 1.22 µM) in comparison to afatinib (IC_50_ 0.0005 µM). The results revealed that oligophenalenone dimers could be used as CDC25B inhibitor candidates [57].

Duclauxin (**120**), talaromycesone B (**122**), bacillisporin G (**127**), and xenoclauxin (**131**) were isolated from anthill soil fungus *Talaromyces* sp. IQ-313. They were evaluated for PTP (protein tyrosine phosphatases) inhibitory potential. They inhibited *h*PTP1B_1-400_ (IC_50_ values ranging from 12.7 to 82.1 µM), in comparison to ursolic acid (IC_50_ 26.6 μM. Compounds **120** and **127** displayed the strongest inhibitory activity (IC_50_ 12.7 and 13.5 µM, respectively) [58]. Five new polar pigments, talauxins E (**132**), I (**133**), L (**134**), Q (**135**), and V (**136**), along with the previously reported 9-demethyl FR-901235 (**55**), O-desmethylfunalenone (**100**), and duclauxin (**120**), were purified from *Talaromyces stipitatus* (Figure 14). Talauxins are unusual heterodimers that are produced from the coupling of **120** with amino acids and are closely related to duclauxamide A (**119**), which was separated from *Penicillium manginii* [56]. They were fully characterized via spectroscopic and X-ray analysis. Compounds **120** and **132** exhibited weak cytotoxic effectiveness (IC_50_ 140 and 70 μM, respectively) versus NS-1 cells, compared to 5-fluorouracil (IC_50_ 4.6 µM) in the resazurin microplate assay, while **132** also had weak antibacterial potential versus *B. subtilis* (IC_50_ 265 μM), compared to clotrimazole (IC_50_ 0.4 μM) [49].

## 4. Human Glucose Transporter 1 (hGLUT1) Inhibitory Activity

### 4.1. Artificial Intelligence (AI)-Based Target Prediction for Phenalenone Derivatives

The human glucose transporter 1 (hGLUT1) is one of 14 members of the GLUT family of integral proteins that are responsible for the facilitative transport of monosaccharides and polyols across the membrane bilayer of eukaryotic cells [68,69]. Structurally, GLUT1 consists of 12 α-helices that are folded into the C-terminal domain and the N-terminal domain, both of which consist of six transmembrane helices [70,71]. Due to its essential role in transporting glucose from the ECM (extracellular matrix) into the cells [72,73] and maintaining the viability of the cells [74], GLUT1 is ubiquitously expressed [74,75]. In many cancer types, the demand for glucose as a source of energy is increased, leading to the increased expression of glucose transporters, including GLUT1 [75,76]. Additionally, the upregulation of GLUT1 expression was found to be mediated by the stimulation of oncogenes [71], while inhibiting GLUT1 activity reduced cell proliferation and apoptosis [71,77,78]. These findings suggest that GLUT1 might be a potential target for cancer therapy [75]. Natural metabolites belonging to diverse classes have been found to possess hGLUT1 inhibition potential, such as resveratrol, phloretin, naringenin, WZB117, cytochalasin B, STF-31, pyrazolopyrimidines, (1H-pyrazol-4-yl)quinoline, and phenylalanine amides [71].

Ligand-based in silico target prediction was performed to choose a suitable target by which to investigate the potential inhibitory activity of the phenalenone derivatives [79,80]. Performing an anatomical-therapeutic chemical (ATC) code and predicting the potential targets for the investigated compounds were carried out using the SuperPred prediction web server [81,82]. From the prediction results, GLUT1 (PDB: **5EQG**) was chosen as a target for the study as it had a high percentage of model accuracy and a very good probability (Table 3). After selecting the target, the docking method was validated by redocking the co-crystalized inhibitor back into the protein crystal structure, then the docking of the listed phenalenones followed. In silico ADMET properties prediction for the listed compounds, along with molecular dynamic (MD) simulation for the two top-scoring derivatives after docking, were performed as well.

### 4.2. In Silico ADMET Properties of Selected Ligands

All 20 phenalenones were prepared for the study by utilizing Schrodinger’s Lig-Prep tool [83]. The 3D (three dimensional) structures of the compounds were generated using the OPLS3 force field setting, with an ionization state at pH 7.0 ± 0.2. After that, ADMET prediction was performed using the QikProp module on Schrodinger’s suite [84]. Table 4 presented the ADMET properties that estimated the phenalenones’ usefulness in terms of their biological functions, drug-likeness, physiochemical properties, and expected toxicity. The ADMET descriptors that are predicted for the derivatives are molecular weight, drug-likeness, dipole moment, total solvent accessible surface area, number of hydrogen bond donors and acceptors, predicted octanol-water partitioning, predicted aqueous solubility, estimated binding to human serum albumin, number of possible metabolites, predicted blood-brain partitioning, percentage of human oral absorption, predicted IC_50_ for inhibiting HERG-K^+^ channels, central nervous system activity, and the reactive functional group number. Most of the predicted values of ADMET descriptors fell within the recommended range.

### 4.3. Ligands and Protein Preparation

The compounds were prepared by converting their structures from 2D to 3D using LigPrep, and their ionization states and tautomeric forms were generated. After energy-minimizing, the 3D structures of the compounds were ready for docking into the crystal structure of GLUT1 (PDB ID: 5EQG). The protein was prepared for docking using the protein preparation wizard, where its crystal structure was minimized and its H-bond network was optimized. In addition, the proper force field was specified, and the protein’s formal charge was calculated after generating the amino acids’ correct ionization states and the missing hydrogen addition.

### 4.4. Grid Box Generation and Molecular Docking

Molecular docking was performed to evaluate the binding modes of the selected compounds inside a protein binding pocket. To do that, a grid box was generated around the protein binding pocket to determine the exact site for the docking in the minimized protein crystal structure, using Maestro’s Receptor-Grid-Generation tool [85]. The docking method was evaluated by re-docking the native inhibitors (PDB ID: **5RE**) back into the crystal structure in which it was co-crystallized. The binding interactions of the re-docked inhibitor are shown in Figure 15. H-bonding was observed between the CO and the NH of the 4-fluorophenylalanine moiety, an adjacent water molecule, and with Glu380, respectively. The second carbonyl group seemed to have H-bonded with Gln161, as well as three nearby water molecules; the phenolic OH acted as both HBD and HBA with water molecules as well.

After validating the docking method, the 3D structures of the minimized phenalenone derivatives were docked into GLUT1. The docking results are presented in Table 5, which shows that, except for derivative **118**, all phenalenones scored higher than the native inhibitor (−11.206 kcal/mol). Derivatives **60** and **64** were on the top of the list, scoring −15.777 and −15.239 kcal/mol, respectively.

**Figure 16:**
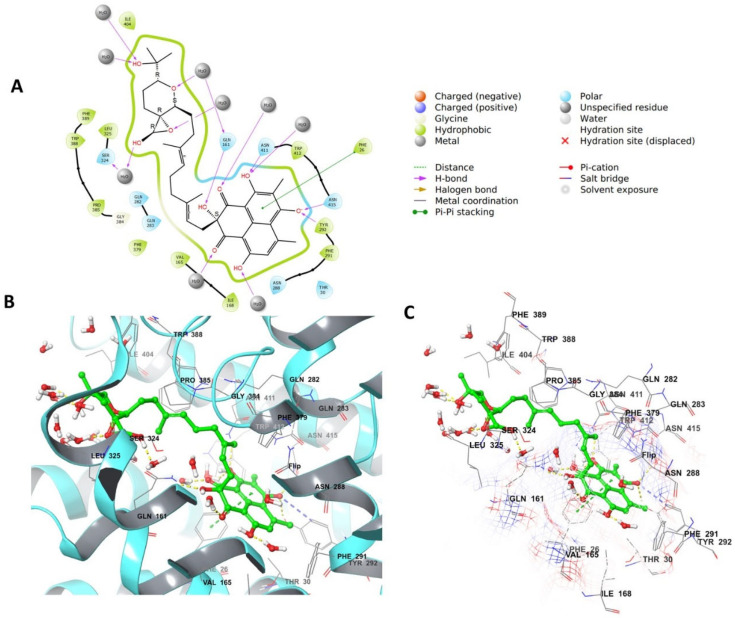
GLUT1-**60** complex after docking. (**A**) 2D view of the binding interactions between **60** complexed with GLUT1 (PDB: 5EQG). (**B**) 3D view of the binding interactions between **60** complexed with GLUT1 (PDB: 5EQG). (**C**) The 3D view of GLUT1-**60** complex with the mesh surface view.

Figure 16 shows the binding interactions of compound **60** after docking. While the Phe26 side chain forms pi-pi stacking with the fused ring system of **60**, several H-bonds are formed between the OH and carbonyl groups of the ring system and the amino acids Gln161, Asn411, Asn415, and Tyr292, as well as forming water bridges with the adjacent water molecules. Additional water bridges are formed with the oxygen and OH groups of the substituted tetrahydropyran at the end of the aliphatic chain.

**Figure 17:**
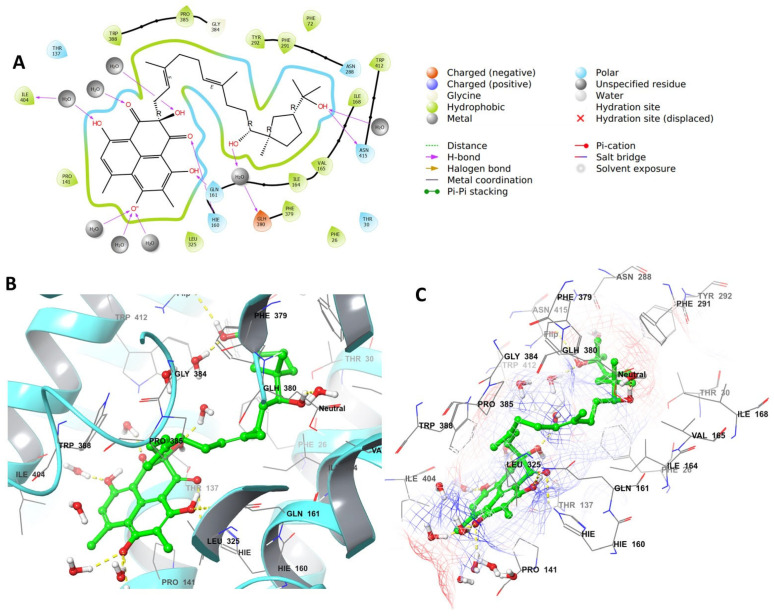
GLUT1-**64** complex after docking. (**A**) 2D view of the binding interactions between compound **64** when complexed with GLUT1 (PDB: 5EQG). (**B**) 3D view of the binding interactions between compound **64** with the amino acids residues of GLUT1 within 3Å radius around the ligand (PDB: 5EQG). (**C**) The 3D view of the GLUT1-**64** complex with the mesh surface indicating the electrostatic potential around the ligand.

As for compound **64**, the carbonyl oxygens and the OH groups of the 3-membered ring system formed several water bridges and H-bonds with the water molecules and His160 and Gln161 side chains. The aliphatic chain and the cyclopentyl moiety interacted with Asn415 and the adjacent water molecules through the OH groups (Figure 17).

### 4.5. Molecular Dynamic Simulation (MD)

**Figure 18:**
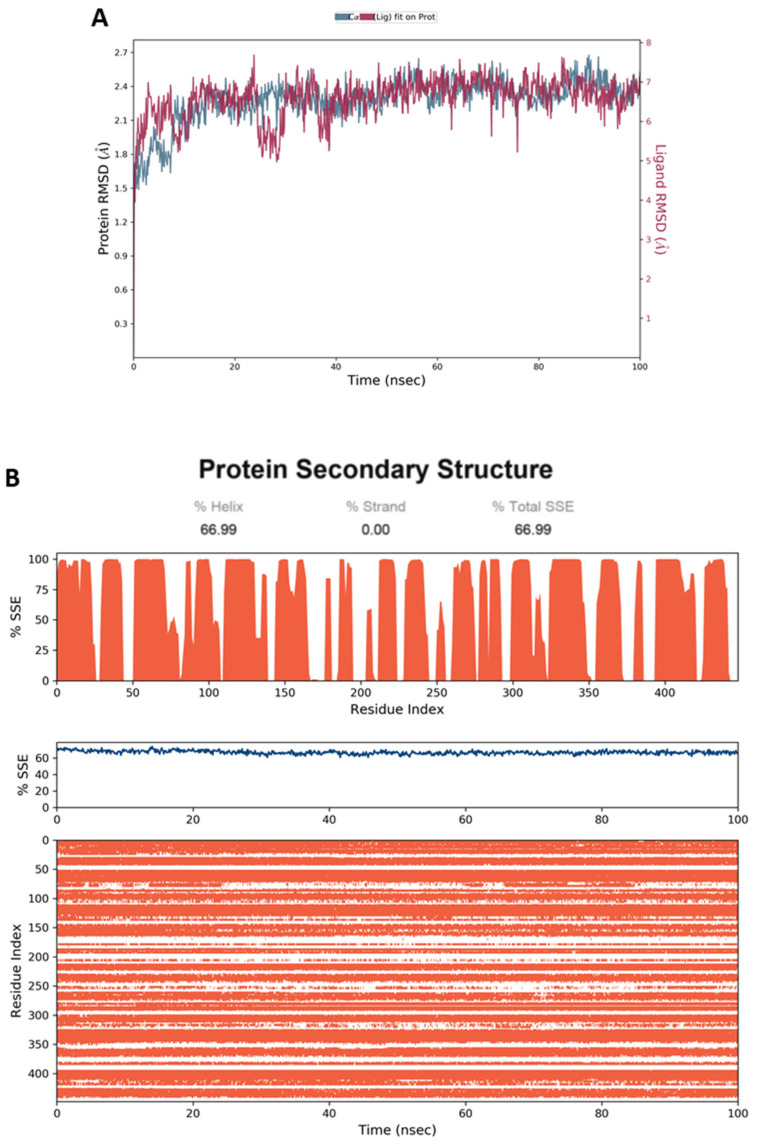
(**A**) RMSD graph for the native inhibitor **5RE**, complexed with the GLUT1 protein (PDB ID: 5EQG). The simulation time (100 ns) confirmed the stability of the complex, with no significant changes in the protein structure. (**B**) Stability of the secondary structure GLUT1 protein (PDB ID: 5EQG) throughout the MD simulation when complexed with **5RE**. Protein secondary structure elements (SSE) were monitored during the simulation. The upper plot presented the SSE distribution by residue index across the protein structure. The middle plot summarized the SSE composition for each trajectory frame throughout the simulation, and the plot at the bottom monitored each residue and its SSE assignment over the simulation time.

**Figure 19:**
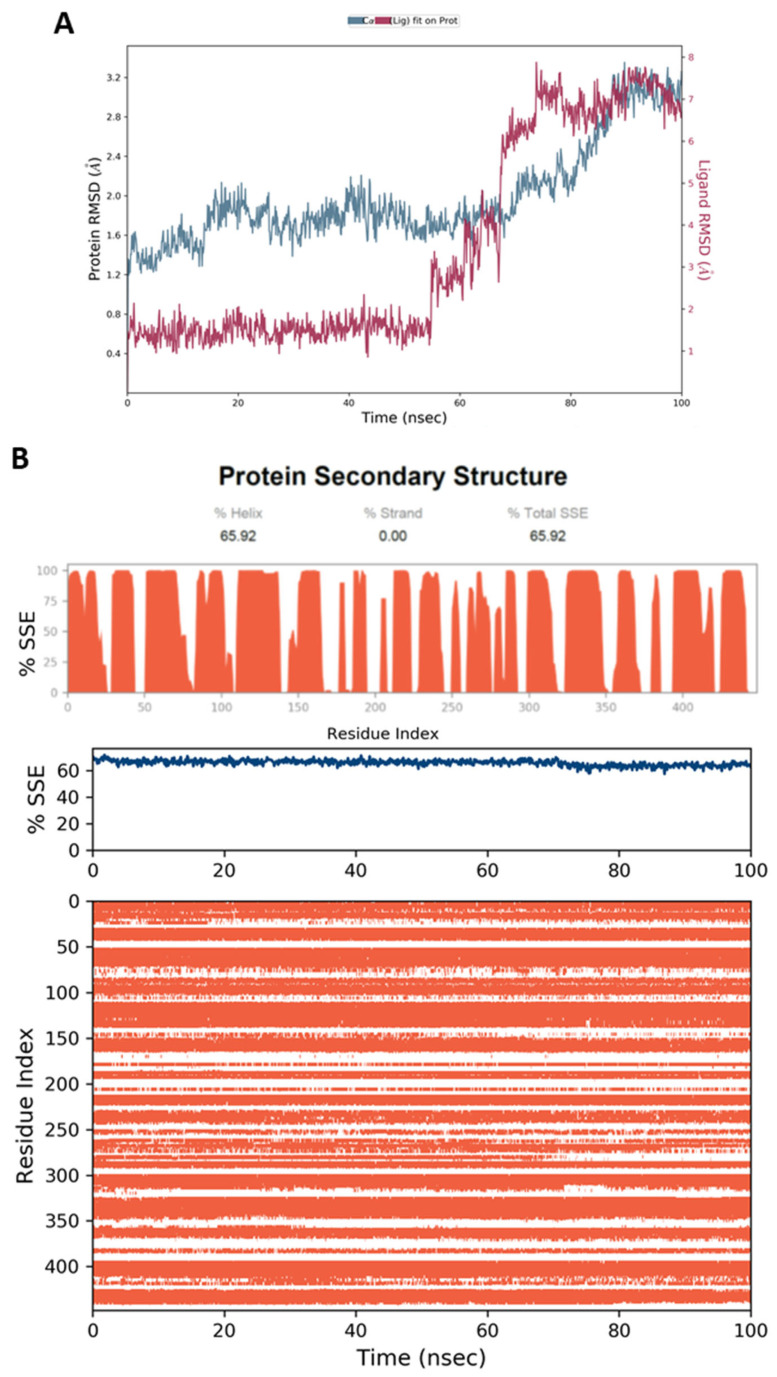
(**A**) RMSD graph for compound **60** when complexed with the GLUT1 protein (PDB ID: 5EQG). The simulation time (100 ns) confirmed the stability of the complex, with no significant changes in the protein structure. (**B**) Stability of the secondary structure GLUT1 protein (PDB ID: 5EQG) throughout the MD simulation when complexed with **60**. The protein secondary structure elements (SSE) were monitored during the simulation. The upper plot presents the SSE distribution according to residue index across the protein structure. The middle plot summarizes the SSE composition for each trajectory frame throughout the simulation, and the plot at the bottom monitors each residue and its SSE assignment over the simulation time.

**Figure 20:**
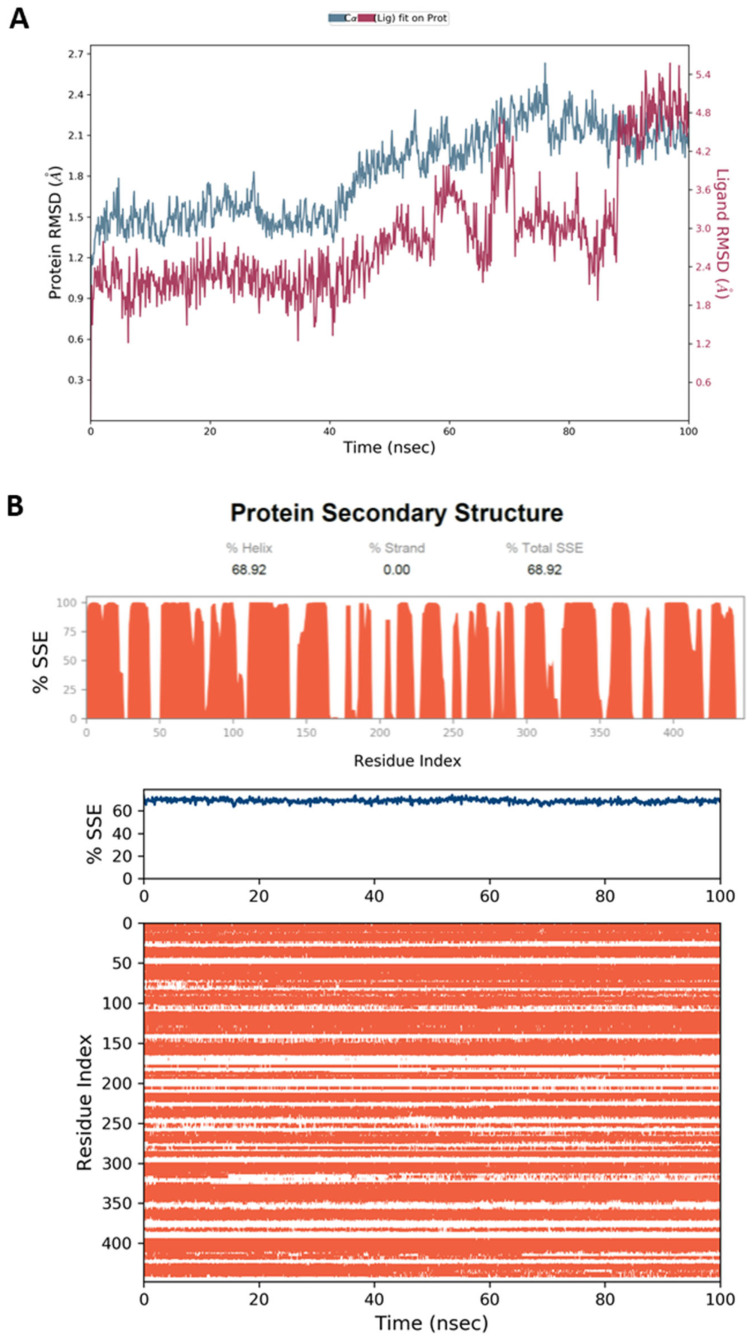
(**A**) RMSD graph for compound **64** when complexed with the GLUT1 protein (PDB ID: 5EQG). The simulation time (100 ns) confirmed the stability of the complex, with no significant changes in the protein structure. (**B**) Stability of the secondary structure of the GLUT1 protein (PDB ID: 5EQG) throughout the MD simulation when complexed with **64**. The protein secondary structure elements (SSE) were monitored during the simulation. The upper plot presents SSE distribution by residue index across the protein structure. The middle plot summarizes the SSE composition for each trajectory frame throughout the simulation, and the plot at the bottom monitors each residue and its SSE assignment over the simulation time.

The MD simulation is a tool that is applied to mimic the physiological environment, to monitor any changes in the protein’s 3D conformation and the binding affinity that might take place during the simulation, and then compare them to the original conformation and affinity of the crystal structure [86]. For that reason, Desmond software [87,88] was used to perform the MD study and evaluate the stability and the binding affinity of the protein-compound complexes at pH 7.0 ± 0.2, over a 100-ns period. The MD was performed only for the two phenalenone derivatives that scored the highest in the docking study, namely, compounds **60** and **64**, as well as the co-crystallized inhibitor, **5RE**. The root mean square deviation (RMSD) of the complexes of compounds and GLUT1 measures the mean change of atoms (of protein and of ligand) at the end of the simulation and compares it to the atoms in their original conformation at 0 ns. The RMSD graph for the GLUT1-**5RE** complex showed that their plots could be laid over each other, indicating high stability of the complex throughout the simulation, and their RMSD values were within the accepted 1–3 Å range (Figure 18A). For the compound **60**-GLUT1 complex, the protein was relatively stable throughout the duration of the experiment, with an RMSD value within the accepted 1–3 Å range. Compound **60** RMSD, however, was stable for about 58% of the time (~1.4–1.5 Å), then the confirmation of the ligand atoms changed drastically afterward, where it re-stabilized again between 5.9 and 7.1 Å by the end of the run. This indicated a change in the 3D conformation of the compound inside the pocket during the run, as the compound adjusted its pose to one with lower free energy. This was most likely due to the presence of many rotatable bonds in the aliphatic side chain (Figure 19A). The RMSD for the GLUT1-**64** complex fell within the acceptable range as well (Figure 20A).

The secondary structure of the protein was scrutinized throughout the MD analysis to ensure that the percentage secondary structure element (%SSE) is intact over the simulation time. Figure 18B demonstrated the integrity of the SSE of the protein when complexed with **5RE**. The top plot showed the distribution of the SSE (α-helices and β-sheets) throughout the protein, represented by the residue index. The middle plot checked the overall %SSE, while the bottom plot assessed each SSE over the course of the simulation. Both plots indicated that the overall %SSE of the protein was maintained, and each SSE was stable over the course of the simulation. A similar result was observed for the GLUT1-**60** and GLUT1-**64** complexes (Figure 19B and Figure 20B, respectively).

**Figure 21:**
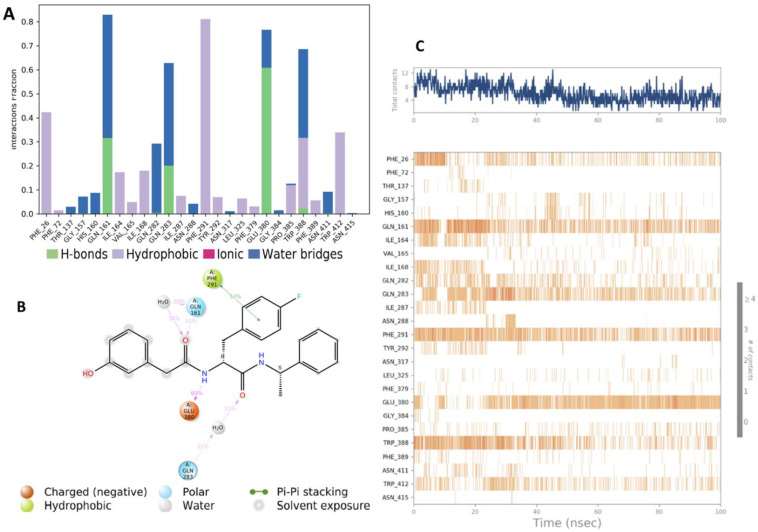
(**A**) The interaction of GLUT1 (PDB: 5EQG) with the native inhibitor **5RE** throughout the simulation. The interactions between the ligand and protein were classified into hydrophobic, hydrogen bond, ionic, and water bridge interactions. The stacked bar charts were normalized over the course of the trajectory: for example, a value of 0.65 suggested that the interaction with the specific amino acid residue was maintained during 65% of the simulation time. Values over 1.0 imply that some protein residue made multiple interactions of the same subtype with the ligand. (**B**) A schematic diagram showed the detailed 2D atomic interactions of **5RE** with GLUT1, occurring for > 30% of the simulation time in the selected trajectory (0 through 100 ns). Interactions with > 100% occurrence meant that those residues may have multiple interactions of a single type with the same ligand atom. (**C**) A timeline representation of the GLUT1-**5RE** interactions shown in (**A**). The panel at the top illustrates the total number of specific interactions that the protein has made with the compound over the course of the trajectory. The panel below illustrates which residues interacted with the ligand in each trajectory frame. The dark or orange color indicated that more than one specific interaction was seen between certain residues and the ligand. # Number of contacts.

The interaction between the test compounds and GLUT1 was also examined by the MD study. Figure 21A illustrates the stacked-bar graph displaying the types of interactions between the pocket residues and the bound ligand. The binding interactions are color-coded in the legend of the figure. The three most prominent observed interactions were as follows. Glu380 achieved H-bonding with the amide nitrogen, with a value of 0.75, while Phe291 formed pi-pi stacking with the 4-fluorophenyl moiety, with a value of 0.8. The carbonyl oxygen of the compound interacted by a water bridge and through H-bonding with Gln161 (normalized value of ~0.83). Additional interactions included that of Gln283, which interacted with the second carbonyl indirectly through a water bridge. Figure 21B shows the 2D view of the binding interactions, depicting interactions that were maintained for at least 30% of the simulation time. Figure 21C is the timeline representation of the stacked-bar graph that presented the interaction pattern of each of the pocket residues of GLUT1 with the **5RE** during the 100 ns of simulation time. The orange color means that there was an interaction, while the darker colors indicate that the residue formed more than one interaction with the ligand.

**Figure 22:**
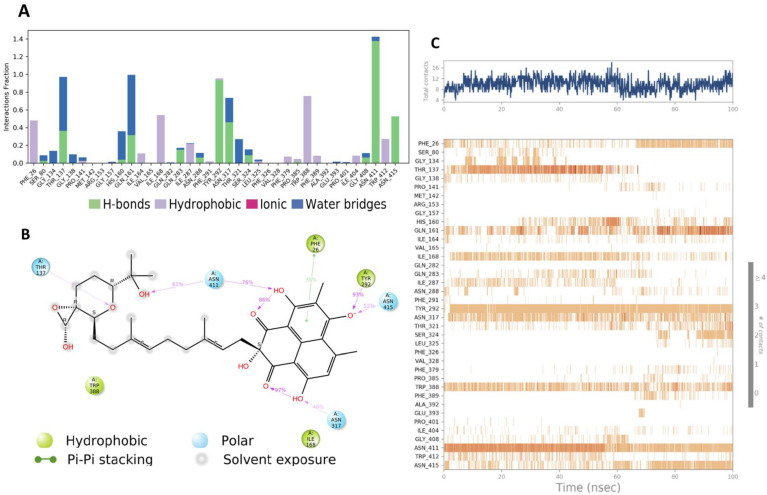
(**A**) The interaction of GLUT1 (PDB: 5EQG) with the native inhibitor **60** throughout the simulation. The interactions between the ligand and protein were classified into hydrophobic, hydrogen bond, ionic, and water bridge interactions. The stacked bar charts were normalized over the course of the trajectory: for example, a value of 0.65 suggested that the interaction with the specific amino acid residue was maintained during 65% of the simulation time. Values over 1.0 implied that some protein residues made multiple interactions of the same subtype with the ligand. (**B**) A schematic diagram showing the detailed 2D atomic interactions of **60** with GLUT1, occurring for > 30% of the simulation time in the selected trajectory (0 through 100 ns). Interactions with > 100% occurrence meant that residues may have multiple interactions of a single type with the same ligand atom. (**C**) A timeline representation of the GLUT1-**60** interactions shown in (**A**). The panel on top illustrates the total number of specific interactions that the protein has made with the compound over the course of the trajectory. The panel below illustrates which residues interacted with the ligand in each trajectory frame. The dark or orange color indicates that more than one specific interaction was made between some residues and the ligand.

The interactions between GLUT1 and **60** included H-bonding and a water bridge with Asn411, yielding a ~1.4 value. In addition, H-bonding and the water bridge contact points were formed with the residues Thr137, Gln161, and Tyr292, along with a hydrophobic interaction with Trp388 (Figure 22A). The 2D view of **60** complexed with GLUT1 showed relatively stable interactions between the compound and the residues Asn411, Trp388, Tyr292, Gln161, and Thr137 throughout the MD run (Figure 22B). From the timeline representation of the stacked bar plot presented in Figure 22C, the same interactions that are illustrated in the stacked bar plot were maintained over 100 ns. However, the interaction of Thr137 with the ligand began strongly and then started disappearing at ~70 ns until the end of the run. This might explain the change in the RMSD plot for 60 after ~58 ns (Figure 19A).

**Figure 23:**
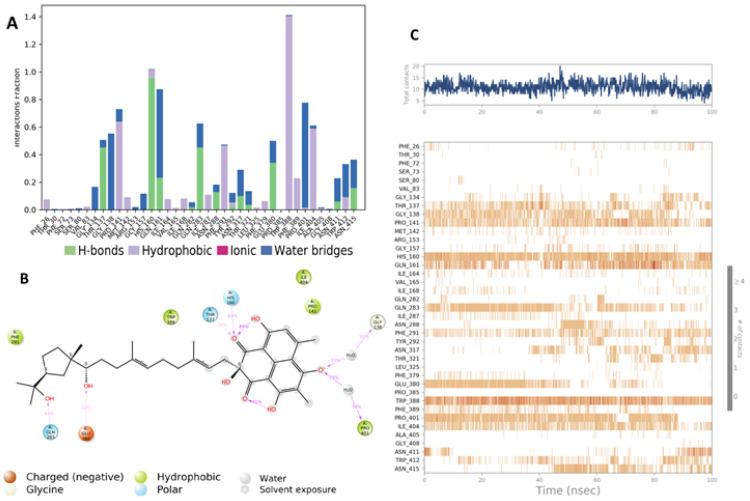
(**A**) The interaction of GLUT1 (PDB: 5EQG) with the native inhibitor 64 throughout the simulation. The interactions between the ligand and protein were classified into hydrophobic, hydrogen bond, ionic, and water bridge interactions. The stacked bar charts were normalized over the course of the trajectory: for example, a value of 0.65 suggested that the interaction with the specific amino acid residue was maintained during 65% of the simulation time. Values over 1.0 implied that some protein residues made multiple interactions of the same subtype with the ligand. (**B**) Schematic diagram showing the detailed 2D atomic interactions of **64** with GLUT1, occurring > 30% of the simulation time in the selected trajectory (0 through 100 ns). Interactions with >100% occurrence meant that residues may have multiple interactions of a single type with the same ligand atom. (**C**) A timeline representation of the GLUT1-**64** interactions shown in (**A**). The panel on top illustrated the total number of specific interactions the protein made with the compound over the course of the trajectory. The panel below illustrates which residues interacted with the ligand in each trajectory frame. The dark or orange color indicates that more than one specific interaction was made between some residues and the ligand.

The amino acid residues involved in binding to derivative **64** included Trp388, Gln238, His160, Gln161, Pro141, and Thr137, with values of between 0.5 and 1.4. A water bridge was observed with Pro401 and Gly138 (Figure 23A). The interactions in the 2D view (Figure 23B) and timeline plot (Figure 23C) agreed with those in the stacked bar graph (Figure 23A).

### 4.6. Material and Methods

#### 4.6.1. ADMET Properties Prediction

The Maestro QikProp Schrodinger module [84] was utilized for the prediction of ADMET properties and drug-likeness for the selected compounds. The properties included absorption, distribution, metabolism, excretion, toxicity, and others.

#### 4.6.2. Preparation of Protein and Ligands PDB Structures

The crystal structure of the GLUT1 with the PDB ID: 5EQG was selected for the experiment because the co-crystalized ligand has a similar structure to the compounds that are to be tested. From the protein databank (PDB), the protein crystal structure 5EQG was downloaded as a PDB file [89] and was then optimized and prepared by the Protein-Preparation wizard of Schrodinger [83,90,91]. Protein preparation and optimization included identifying the bond order for the known HET groups and untemplated residues, adding hydrogen, breaking bonds to metals, adding zero-order bonds between metals and adjacent atoms, and correcting the formal charges to metals and the nearby atoms. Water molecules further than 5 Å from HET groups were removed from the structure of HET groups, and the disulfide bonds were re-generated. Ligands were prepared using the Lig-Prep tool [83], which involved the generation of metal HET states and cofactors at pH 7 ± 2.0. Additionally, the optimization of hydrogen bonds at pH 7.0 using PROPKA [92], the removal of water molecules of >3 Å from HET groups, and applying restrained minimization using the OPLS4 force field were performed.

#### 4.6.3. Grid Generation and Docking

Glide’s Receptor-Grid-Generation tool [85] was used to generate a grid box around the co-crystalized inhibitor **5RE** in the binding site of the protein PDB: 5EQG. The docking of the phenalenones was performed inside this box. The non-polar atoms were set for a van der Waals (VdW) radii scaling factor of 1.0 and the cut-off of partial charge was 0.25. Schrodinger’s Ligand Docking tool was used to perform the docking procedure [85,93]. The docking protocol was set as standard precision (SP), while the ligand sampling method was flexible. The default settings were used for other parameters.

#### 4.6.4. MD Simulation

MD simulation experiments were performed using the Schrodinger suite [87,88]. The selected protein-compound complexes were obtained from the docking results and were tuned through the “System-Builder” tool. TIP3P was selected as the solvent mode, and the chosen box shape was the orthorhombic shape. Na ions were added to neutralize the system, and the box dimensions were 10 Å. The duration of the MD simulations was 100 ns per trajectory, and the number of atoms, temperature, and pressure were kept constant (NPT ensemble). Conversely, the temperature was set at 300.0 K, the pressure was set at 1.01325 bar, and the force field was OPLS4.

## 5. Conclusions

**Figure 24:**
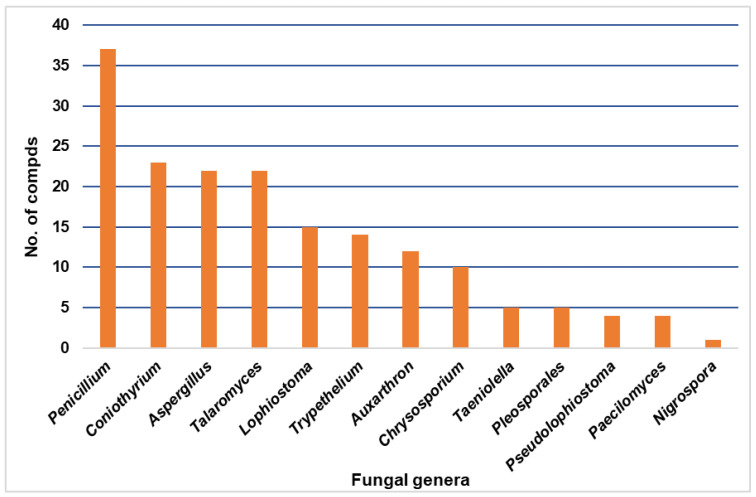
The numbers of phenalenones isolated from the different fungal genera.

Fungi-derived metabolites possess substantial medicinal values and a large structural diversity that can provide an untapped potential for drug candidates and medications. This review summarized 139 fungal phenalenone derivatives and their biosynthesis and biological activities, reported from 2014 until August 2021. Most of them are mainly identified from *Penicillium* (37 compounds), *Coniothyrium* (23 compounds), *Aspergillus* (22 compounds), and *Talaromyces* (22 compounds) (Figure 24).

**Figure 25:**
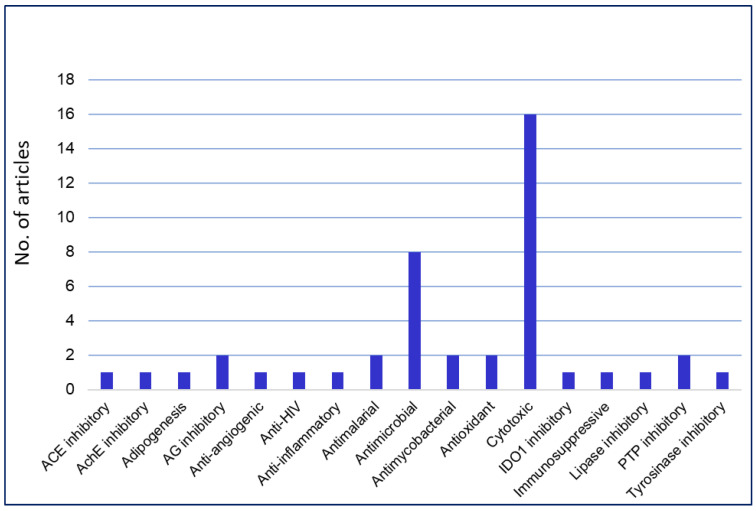
Biological activities of isolated phenalenones and the number of articles.

Fungal phenalenones were derived from polyketide precursors that underwent different cyclization and tailoring reactions, leading to extreme structural diversity and high complexity. Hence, searching for diverse biosynthetic pathways for the various phenalenone derivatives will be a future challenge and offer an interesting research field for natural product researchers. These metabolites have been isolated and purified using various chromatographic tools, such as SiO_2_, Sephadex LH-20, preparative TLC, ODS, and HPLC, as well as preparative HSCCC-guided HPLC-HRESIMS, LC-MS-guided, and UV-HPLC guided analyses. Most of the separated phenalenones possess unique and unprecedented functionality or ring systems. Their configuration was assigned using various tools and experiments, such as X-rays, CD, ECD calculation, specific rotations, and chemical modifications, as well as the NOESY, NOEDIFF, and GIAO NMR shift calculations. They were evaluated for various activities, such as cytotoxic, antimalarial, antimycobacterial, anti-inflammatory, anti-angiogenic, immunosuppressive, and antioxidant properties, as well as IDO1, α-glucosidase (AG), ACE, IDO1, tyrosinase, and PTP inhibition (Figure 25). Some of the reported derivatives possessed powerful activities greater than in the used controls, such as anti-HIV (e.g., **71** and **74)**, immunosuppressive (e.g., **17**), anti-tumor (e.g., **120**, **125**, **129**, and **131**), and antibacterial behavior (e.g., **24**, **31**, **69**, **83**, and **85**), in addition to tyrosinase (e.g., **1**–**3**), α-glucosidase (e.g., **29**, **34**, **46**, and **47)**, pancreatic lipase (e.g., **29** and **34**), PTP (e.g., **101**, **120**, and **127**), and ACE (e.g., **104**) inhibitory behavior. Further in vivo and clinical studies should be conducted to validate these bioactivities. Conversely, many of the newly separated phenalenones possessed weak or no bioactivity, which represents a common problem for the study of the natural product. This could be due to the insufficient amounts of the new compounds and the lack of effective bioactivity screening methods. Cancer is one of the most significant worldwide health concerns and there is a continuous need for developing new targets for treating this disease. GLUT1 substantially increases the uptake of glucose into the cytoplasm and is over-expressed in various tumor cells. Therefore, it is likely to be a potential target for treating cancer. Based on the in silico studies, such as molecular docking, ADMET characteristics predication, and MD, some phenalenones were found to possess remarkable capacity as GLUT1 inhibitors; therefore, they could be potential leads for cancer treatment. It is noteworthy that the described results in this work are reported for the first time for this class of fungal metabolites and undoubtedly represent a substantial contribution in terms of further investigation, as well as in vitro and in vivo evaluations.

Discovering bioactive phenalenones for drug use can be accelerated by applying and developing new technology, such as the methods of biosynthetic gene cluster (BGCs) activation for mining hidden new compounds [94], the use of metabolomic and genomic approaches [95], and modern machine deep learning techniques to discover the structurally distinct bioactive molecules [96,97]. Finally, we believe that the therapeutic potential and chemical diversity of the fungal phenalenones after more in-depth research will provide medicinal chemists and biologists with a more promising sustainable treasure trove for drug discovery.

## Figures and Tables

**Table 1:** List of fungal phenalenones (fungal source, host, and place of origin).

Compound Name	Mol. Wt.	Mol. Formula	Fungal Source	Host (Part, Family)	Place	Ref.
Paecilomycone A (**1**)	288	C_15_H_12_O_6_	*Paecilomyces gunnii*	Culture	China	[32]
Paecilomycone B (**2**)	286	C_15_H_10_O_6_	*Paecilomyces gunnii*	Culture	China	[32]
Paecilomycone C (**3**)	287	C_15_H_13_NO_5_	*Paecilomyces gunnii*	Culture	China	[32]
Myeloconone A (**4**)	286	C_16_H_14_O_5_	*Paecilomyces gunnii*	Culture	China	[32]
Aspergillussanone A (**5**)	540	C_31_H_40_O_8_	*Aspergillus* sp. PSU-RSPG185	Soil	Surat Thani, Thailand	[33]
Aspergillussanone B (**6**)	540	C_31_H_40_O_8_	*Aspergillus* sp. PSU-RSPG185	Soil	Surat Thani, Thailand	[33]
Peniciherqueinone (**7**)	388	C_20_H_20_O_8_	*Penicillium herquei* PSU-RSPG93	Soil	Surat Thani, Thailand	[34]
*ent*-Peniciherqueinone (**8**)	388	C_20_H_20_O_8_	*Penicillium* sp.	Marine sediment	Gagudo, Korea	[35]
Herqueinone (**9**)	372	C_20_H_20_O_7_	*Penicillium herquei* PSU-RSPG93	Soil	Surat Thani, Thailand	[34]
			*Penicillium* sp.	Marine sediment	Gagudo, Korea	[35]
			*Penicillium* sp. G1071	Mushroom fruiting body	Freshwater stream, Hebron, Connecticut, USA	[36]
Norherqueinone (**10**)	358	C_19_H_18_O_7_	*Penicillium* sp. G1071	Mushroom fruiting body	Freshwater stream, Hebron, Connecticut, USA	[36]
12-Hydroxynorherqueinone (**11**)	374	C_19_H_18_O_8_	*Penicillium* sp.	Marine sediment	Gagudo, Korea	[35]
Isoherqueinone (**12**)	372	C_20_H_20_O_7_	*Penicillium* sp.	Marine sediment	Gagudo, Korea	[35]
*ent*-Isoherqueinone (**13**)	372	C_20_H_20_O_7_	*Penicillium* sp.	Marine sediment	Gagudo, Korea	[35]
Deoxyherqueinone (**14**)	356	C_20_H_20_O_6_	*Penicillium herquei* PSU-RSPG93	Soil	Surat Thani, Thailand	[34]
Oxopropylisoherqueinone A (**15**)	414	C_22_H_22_O_8_	*Penicillium* sp.	Marine sediment	Gagudo, Korea	[35]
Oxopropylisoherqueinone B (**16**)	414	C_22_H_22_O_8_	*Penicillium* sp.	Marine sediment	Gagudo, Korea	[35]
Diketoacetonylphenalenone = Acetone adduct of a triketone (**17**)	398	C_22_H_22_O_7_	*Penicillium* sp.	Marine sediment	Gagudo, Korea	[35]
Acetone adduct of antatrovenetinone (**18**)	398	C_22_H_22_O_7_	*Penicillium herquei* PSU-RSPG93	Soil	Surat Thani, Thailand	[34]
			*Coniothyrium cereale*	*Enteromorpha* sp. (Algae, Ulvaceae)	Fehmarn, Baltic Sea, Germany	[37]
			*Penicillium herquei* FT729	Volcanic soil sample	Big Island, Hawaii	[38]
Antatrovenetinone (**19**)	340	C_19_H_16_O_6_	*Coniothyrium cereale*	*Enteromorpha* sp. (Algae, Ulvaceae)	Fehmarn, Baltic Sea, Germany	[37]
Sclerodin (**20**)	328	C_18_H_16_O_6_	*Penicillium herquei* PSU-RSPG93	Soil	Surat Thani, Thailand	[34]
(+)-Sclerodin (**21**)	328	C_18_H_16_O_6_	*Penicillium* sp. ZZ901	*Scapharca broughtonii* (Schrenck blood clam, Arcidae)	Sea shoal, East China, Zhoushan Archipelago, China	[39]
			*Chrysosporium lobatum* TM-237-S5	*Acanthella cavernosa* (Sponge, Dictyonellidae)	Mesophotic reef in Eilat, Dekel Beach, Gulf of Aqaba, Israel	[40]
			*Trypethelium eluteriae* Sprengl	*Anacardium occidentale* (Bark, Anacardiaceae)	Vadanemmeli, Kanchipuram, Tamil Nadu, India	[41]
(−)-Sclerodin (**22**)	328	C_18_H_16_O_6_	*Lophiostoma bipolare* BCC25910	Mangrove wood	Beach in Haad Wanakorn National Park, Prachuap Khiri Khan, Thailand	[42]
			*Penicillium* sp.	Marine sediment	Gagudo, Korea	[35]
			*Taeniolella* sp. BCC31839	Wood of mangrove forest (Poaceae)	Bangkok, Thailand	[43]
			*Coniothyrium cereale*	*Enteromorpha* sp. (Algae, Ulvaceae)	Fehmarn, Baltic Sea, Germany	[20]
(+)-8-Hydroxyslerodin (**23**)	344	C_18_H_16_O_7_	*Chrysosporium lobatum* TM-237-S5	*Acanthella cavernosa* (Sponge, Dictyonellidae)	Mesophotic reef in Eilat, Dekel Beach, Gulf of Aqaba, Israel	[40]
(−)-Sclerodinol (**24**)	344	C_18_H_16_O_7_	*Pleosporales* sp. HDN1811400	Sediment sample	Fildes Peninsula	[44]
			*Coniothyrium cereale*	*Enteromorpha* sp. (Algae, Ulvaceae)	Fehmarn, Baltic Sea, Germany	[20]
(−)-Bipolaride D (**25**)	342	C_19_H_18_O_6_	*Lophiostoma bipolare* BCC25910	Mangrove wood	Beach in Haad Wanakorn National Park, Prachuap Khiri Khan, Thailand	[42]
			*Taeniolella* sp. BCC31839	Wood of mangrove forest (Poaceae)	Bangkok, Thailand	[43]
Bipolarol C (**26**)	344	C_18_H_16_O_7_	*Lophiostoma bipolare* BCC25910	Mangrove wood	Beach in Haad Wanakorn National Park, Prachuap Khiri Khan, Thailand	[42]
4-Hydroxysclerodin (**27**)	346	C_18_H_18_O_7_	*Penicillium* sp.	Marine sediment	Gagudo, Korea	[35]
(+)-Sclerodione (**28**)	312	C_18_H_16_O_5_	*Penicillium* sp. ZZ901	*Scapharca broughtonii* (Schrenck blood clam, Arcidae)	Sea shoal, East China, Zhoushan Archipelago, China	[39]
(−)-Sclerodione (**29**)	312	C_18_H_16_O_5_	*Pseudolophiostoma* sp. MFLUCC 17-2081	*Clematis fulvicoma* (Ranunculaceae)	Chiang Rai, Thailand	[45]
			*Taeniolella* sp. BCC31839	Wood of mangrove forest (Poaceae)	Bangkok, Thailand	[43]
			*Coniothyrium cereale*	*Enteromorpha* sp. (Algae, Ulvaceae)	Fehmarn, Baltic Sea, Germany	[20]
(−)-Bipolaride B (**30**)	326	C_19_H_18_O_5_	*Lophiostoma bipolare* BCC25910	Mangrove wood	Beach in Haad Wanakorn National Park, Prachuap Khiri Khan, Thailand	[42]
			*Taeniolella* sp. BCC31839	Wood of mangrove forest (Poaceae)	Bangkok, Thailand	[43]
Peniciphenalenin H (**31**)	328	C_18_H_16_O_6_	*Pleosporales* sp. HDN1811400	Sediment sample	Fildes Peninsula	[44]
Bipolarol A (**32**)	342	C_19_H_18_O_6_	*Lophiostoma bipolare* BCC25910	Mangrove wood	Beach in Haad Wanakorn National Park, Prachuap Khiri Khan, Thailand	[42]
(+)-Scleroderolide (**33**)	328	C_18_H_16_O_6_	*Penicillium* sp. ZZ901	*Scapharca broughtonii* (Schrenck blood clam, Arcidae)	Sea shoal, East China, Zhoushan Archipelago, China	[39]
			*Chrysosporium lobatum* TM-237-S5	*Acanthella cavernosa* (Sponge, Dictyonellidae)	Mesophotic reef in Eilat, Dekel Beach, Gulf of Aqaba, Israel	[40]
			*Penicillium herquei* MA-370	Rhizospheric soil of the mangrove plant *Rhizophora mucronata* (Rhizophoraceae)	Hainan Island, China	[46]
(−)-Scleroderolide (**34**)	328	C_18_H_16_O_6_	*Lophiostoma bipolare* BCC25910	Mangrove wood	Beach in Haad Wanakorn National Park, Prachuap Khiri Khan, Thailand	[42]
			*Penicillium* sp.	Marine sediments	Gagudo, Korea	[35]
			*Pseudolophiostoma* sp. MFLUCC 17-2081	*Clematis fulvicoma* (Ranunculaceae)	Chiang Rai, Thailand	[45]
			*Taeniolella* sp. BCC31839	Wood of mangrove forest (Poaceae)	Bangkok, Thailand	[43]
			*Penicillium herquei* MA-370	Rhizospheric soil of the mangrove plant *Rhizophora mucronata* (Rhizophoraceae)	Hainan Island, China	[46]
			*Coniothyrium cereale*	*Enteromorpha* sp. (Algae, Ulvaceae)	Fehmarn, Baltic Sea, Germany	[20]
(+)-8-Hydroxyscleroderolide (**35**)	344	C_18_H_16_O_7_	*Chrysosporium lobatum* TM-237-S5	*Acanthella cavernosa* (Sponge, Dictyonellidae)	Mesophotic reef in Eilat, Dekel Beach, Gulf of Aqaba, Israel	[40]
(−)-Bipolaride A (**36**)	342	C_19_H_18_O_6_	*Lophiostoma bipolare* BCC25910	Mangrove wood	Beach in Haad Wanakorn National Park, Prachuap Khiri Khan, Thailand	[42]
(−)-Cereolactone = (−)-7,8-Dihydro-3,6-dihydroxy-1,7,7,8-tetramethyl-5H-furo-[2′,3′:5,6]naphtho-[1,8-*bc*]furan-5-one (**37**)	300	C_17_H_16_O_5_	*Penicillium herquei* PSU-RSPG93	Soil	Surat Thani, Thailand	[34]
			*Chrysosporium lobatum* TM-237-S5	*Acanthella cavernosa* (Sponge, Dictyonellidae)	Mesophotic reef in Eilat, Dekel Beach, Gulf of Aqaba, Israel	[40]
			*Coniothyrium cereale*	*Enteromorpha* sp. (Algae, Ulvaceae)	Fehmarn, Baltic Sea, Germany	[20]
(R)-6-Hydroxy-3-methoxy-1,7,7,8-tetramethyl-7,8-dihydro-5H-naphtho[1,2-b:5,4-b′c′]difuran-5-one (**38**)	314	C_18_H_18_O_5_	*Trypethelium eluteriae*	*Anacardium occidentale* (Bark, Anacardiaceae)	Vadanemmeli, Kanchipuram, Tamil Nadu, India	[41]
(−)-Bipolaride E (**39**)	314	C_18_H_18_O_5_	*Lophiostoma bipolare* BCC25910	Mangrove wood	Beach in Haad Wanakorn National Park, Prachuap Khiri Khan, Thailand	[42]
Bipolarol B (**40**)	344	C_19_H_20_O_6_	*Lophiostoma bipolare* BCC25910	Mangrove wood	Beach in Haad Wanakorn National Park, Prachuap Khiri Khan, Thailand	[42]
Bipolarol D (**41**)	330	C_18_H_18_O_6_	*Lophiostoma bipolare* BCC25910	Mangrove wood	Beach in Haad Wanakorn National Park, Prachuap Khiri Khan, Thailand	[42]
Bipolaride C (**42**)	328	C_19_H_20_O_5_	*Lophiostoma bipolare* BCC25910	Mangrove wood	Beach in Haad Wanakorn National Park, Prachuap Khiri Khan, Thailand	[42]
2,5,7-Trihydroxy-4-(3′-methylbut-2′-en-1′-yl) oxy-2*H*-naphtho [1,8-*bc*] furan-9-one (**43**)	302	C_16_H_14_O_6_	*Nigrospora sphaerica*	*Xylocarpus granatum* (Mangrove plant, Meliaceae)	Intertidal zone, China Sea, Huanghai, China	[47]
Rousselianone A = 4,9-Dihydroxy-6-methyl-7-((3-methylbut-2-en-1-yl)oxy) -1*H*-phenalene-1,2,3-trione (**44**)	340	C_19_H_16_O_6_	*Coniothyrium cereale*	*Enteromorpha* sp. (Algae, Ulvaceae)	Fehmarn, Baltic Sea, Germany	[20,37]
Flaviphenalenone A (**45**)	442	C_24_H_26_O_8_	*Aspergillus flavipes* PJ03-11	Wetland mud	Panjin Red Beach National Nature Reserve, Liaoning, China	[31]
Flaviphenalenone B (**46**)	372	C_20_H_20_O_7_	*Aspergillus flavipes* PJ03-11	Wetland mud	Panjin Red Beach National Nature Reserve, Liaoning, China	[31]
Flaviphenalenone C (**47**)	372	C_20_H_20_O_7_	*Aspergillus flavipes* PJ03-11	Wetland mud	Panjin Red Beach National Nature Reserve, Liaoning, China	[31]
Rousselianone A′ = Acetone adduct of 4,9-dihydroxy-6-methyl-7-((3-methylbut-2-en-1-yl)oxy)-1*H*-phenalene-1,2,3-trione (**48**)	398	C_22_H_22_O_7_	*Coniothyrium cereale*	*Enteromorpha* sp. (Algae, Ulvaceae)	Fehmarn, Baltic Sea, Germany	[37]
			*Coniothyrium cereale*	*Enteromorpha* sp. (Algae, Ulvaceae)	Fehmarn, Baltic Sea, Germany	[20]
Auxarthrone A (**49**)	358	C_19_H_18_O_7_	*Auxarthron pseudauxarthron* TTI-0363	*Sylvilagus audubonii* (Desert rabbit dung, Leporidae)	U.S. Route 385, Marathon, and the north entrance of Big Bend National Park, Brewster, Texas, USA	[48]
Auxarthrone B (**50**)	358	C_19_H_18_O_7_	*Auxarthron pseudauxarthron* TTI-0363	*Sylvilagus audubonii* (Desert rabbit dung, Leporidae)	U.S. Route 385, Marathon, and the north entrance of Big Bend National Park, Brewster, Texas, USA	[48]
Auxarthrone D (**51**)	372	C_20_H_20_O_7_	*Auxarthron pseudauxarthron* TTI-0363	*Sylvilagus audubonii* (Desert rabbit dung, Leporidae)	U.S. Route 385, Marathon, and the north entrance of Big Bend National Park, Brewster, Texas, USA	[48]
Auxarthrone C (**52**)	340	C_19_H_16_O_6_	*Auxarthron pseudauxarthron* TTI-0363	*Sylvilagus audubonii* (Desert rabbit dung, Leporidae)	U.S. Route 385, Marathon, and the north entrance of Big Bend National Park, Brewster, Texas.	[48]
Auxarthrone E (**53**)	354	C_20_H_18_O_6_	*Auxarthron pseudauxarthron* TTI-0363	*Sylvilagus audubonii* (Desert rabbit dung, Leporidae)	U.S. Route 385, Marathon, and the north entrance of Big Bend National Park, Brewster, Texas, USA	[48]
FR-901235 (**54**)	344	C_18_H_16_O_7_	*Auxarthron pseudauxarthron* TTI-0363	*Sylvilagus audubonii* (Desert rabbit dung, Leporidae)	U.S. Route 385, Marathon, and the north entrance of Big Bend National Park, Brewster, Texas, USA	[48]
9-Demethyl FR-901235 (**55**)	330	C_17_H_14_O_7_	*Talaromyces stipitatus*	Soil	Collaroy, New South Wales, Australia	[49]
(10*S*)-6-amino-3,6,8-trihydroxy-1,9,9,10-tetramethyl-9,10-dihydro-5H-furo[2′,3′:5,6]naphtho[1,8-*bc*]oxepine-5,7(6*H*)-dione (**56**)	373	C_19_H_19_NO_7_	*Coniothyrium cereale*	*Enteromorpha* sp. (Algae, Ulvaceae)	Fehmarn, Baltic Sea, Germany	[37]
(*S*)-3,8-Dihydroxy-6-imino-1,9,9,10-tetramethyl-9,10-dihydro-5H-furo[2′,3′:5,6]naphtho[1,8-*bc*]oxepine -5,7(6*H*)-dione (**57**)	355	C_19_H_17_NO_6_	*Coniothyrium cereale*	*Enteromorpha* sp. (Algae, Ulvaceae)	Fehmarn, Baltic Sea, Germany	[37]
*S*-Dehydroazasirosterol (**58**)		C_47_H_59_NO_6_	*Coniothyrium cereale*	*Enteromorpha* sp. (Algae, Ulvaceae)	Fehmarn, Baltic Sea, Germany	[37]
Conio-azasterol (**59**)	733	C_47_H_59_NO_6_	*Coniothyrium cereale*	*Enteromorpha* sp. (Algae, Ulvaceae)	Fehmarn, Baltic Sea, Germany	[37]
Aspergillussanone C (**60**)	624	C_35_H_44_O_10_	*Aspergillus* sp.	Pinellia ternate (Tuber, Araceae)	Suburb of Nanjing, Jiangsu, China	[50]
Aspergillussanone D (**61**)	592	C_35_H_44_O_8_	*Aspergillus* sp.	*Pinellia ternate* (Tuber, Araceae)	Suburb of Nanjing, Jiangsu, China	[50]
Aspergillussanone E (**62**)	592	C_35_H_44_O_8_	*Aspergillus* sp.	*Pinellia ternate* (Tuber, Araceae)	Suburb of Nanjing, Jiangsu, China	[50]
Aspergillussanone F (**63**)	594	C_35_H_46_O_8_	*Aspergillus* sp.	*Pinellia ternate* (Tuber, Araceae)	Suburb of Nanjing, Jiangsu, China	[50]
Aspergillussanone G (**64**)	610	C_35_H_46_O_9_	*Aspergillus* sp.	*Pinellia ternate* (Tuber, Araceae)	Suburb of Nanjing, Jiangsu, China	[50]
Aspergillussanone H (**65**)	606	C_35_H_42_O_9_	*Aspergillus* sp.	*Pinellia ternate* (Tuber, Araceae)	Suburb of Nanjing, Jiangsu, China	[50]
Aspergillussanone I (**66**)	634	C_38_H_50_O_8_	*Aspergillus* sp.	*Pinellia ternate* (Tuber, Araceae)	Suburb of Nanjing, Jiangsu, China	[50]
Aspergillussanone J (**67**)	666	C_38_H_50_O_10_	*Aspergillus* sp.	*Pinellia ternate* (Tuber, Araceae)	Suburb of Nanjing, Jiangsu, China	[50]
Aspergillussanone K (**68**)	652	C_37_H_48_O_10_	*Aspergillus* sp.	*Pinellia ternate* (Tuber, Araceae)	Suburb of Nanjing, Jiangsu, China	[50]
Aspergillussanone L (**69**)	594	C_35_H_46_O_8_	*Aspergillus* sp.	*Pinellia ternate* (Tuber, Araceae)	Suburb of Nanjing, Jiangsu, China	[50]
(*S*)-2-((*S,*2*E,*6*E,*10*Z*)-14,15-Dihydroxy-11-(hydroxymethyl)-3,7,15-trimethylhexadeca-2,6,10-trien-1-yl)-2,4,6,9-tetrahydroxy- 5,7-dimethyl-1*H*-phenalene-1,3(2*H*)-dione (**70**)	610	C_35_H_46_O_9_	*Aspergillus* sp.	*Pinellia ternate* (Tuber, Araceae)	Suburb of Nanjing, Jiangsu, China	[50]
Asperphenalenone A (**71**)	576	C_35_H_44_O_7_	*Aspergillus* sp. CPCC 400735	*Kadsura longipedunculata* (Schisandraceae)	Leishan, in Guizhou province, China	[51]
Asperphenalenone B (**72**)	624	C_35_H_44_O_10_	*Aspergillus* sp. CPCC 400735	*Kadsura longipedunculata* (Schisandraceae)	Leishan, in Guizhou province, China	[51]
Asperphenalenone C (**73**)	624	C_35_H_44_O_10_	*Aspergillus* sp. CPCC 400735	*Kadsura longipedunculata* (Schisandraceae)	Leishan, in Guizhou province, China	[51]
Asperphenalenone D (**74**)	654	C_37_H_50_O_10_	*Aspergillus* sp. CPCC 400735	*Kadsura longipedunculata* (Schisandraceae)	Leishan, in Guizhou province, China	[51]
Asperphenalenone E (**75**)	610	C_35_H_46_O_9_	*Aspergillus* sp. CPCC 400735	*Kadsura longipedunculata* (Schisandraceae)	Leishan, in Guizhou province, China	[51]
Peniciphenalenin A (**76**)	328	C_18_H_16_O_6_	*Penicillium* sp. ZZ901	*Scapharca broughtonii* (Schrenck blood clam, Arcidae)	Sea shoal, East China, Zhoushan Archipelago, China	[39]
Peniciphenalenin B (**77**)	404	C_20_H_20_O_9_	*Penicillium* sp. ZZ901	*Scapharca broughtonii* (Schrenck blood clam, Arcidae)	Sea shoal, East China, Zhoushan Archipelago, China	[39]
Peniciphenalenin C (**78**)	418	C_21_H_22_O_9_	*Penicillium* sp. ZZ901	*Scapharca broughtonii* (Schrenck blood clam, Arcidae)	Sea shoal, East China, Zhoushan Archipelago, China	[39]
Peniciphenalenin D (**79**)	316	C_17_H_16_O_6_	*Penicillium* sp. ZZ901	*Scapharca broughtonii* (Schrenck blood clam, Arcidae)	Sea shoal, East China, Zhoushan Archipelago, China	[39]
			*Chrysosporium lobatum* TM-237-S5	*Acanthella cavernosa* (Sponge, Dictyonellidae)	Mesophotic reef in Eilat, Dekel Beach, Gulf of Aqaba, Israel	[40]
Peniciphenalenin E, Peniciphenalenin Ea (**80**)	300	C_17_H_16_O_5_	*Penicillium* sp. ZZ901	*Scapharca broughtonii* (Schrenck blood clam, Arcidae)	Sea shoal, East China, Zhoushan Archipelago, China	[39]
Peniciphenalenin F (**81**)	300	C_17_H_16_O_5_	*Penicillium* sp. ZZ901	*Scapharca broughtonii* (Schrenck blood clam, Arcidae)	Sea shoal, East China, Zhoushan Archipelago, China	[39]
(−)-Peniciphenalenin F (**82**)	300	C_17_H_16_O_5_	*Chrysosporium lobatum* TM-237-S5	*Acanthella cavernosa* (Sponge, Dictyonellidae)	Mesophotic reef in Eilat, Dekel Beach, Gulf of Aqaba, Israel	[40]
Peniciphenalenin G (**83**)	364	C_18_H_17_ClO_6_	*Pleosporales* sp. HDN1811400	Sediment	Fildes Peninsula	[44]
Peniciphenalenin I (**84**)	328	C_18_H_16_O_6_	*Pleosporales* sp. HDN1811400	Sediment	Fildes Peninsula	[44]
Coniosclerodione (**85**)	312	C_18_H_16_O_5_	*Pleosporales* sp. HDN1811400	Sediment	Fildes Peninsula	[44]
			*Coniothyrium cereale*	*Enteromorpha* sp. (Algae, Ulvaceae)	Fehmarn, Baltic Sea, Germany	[20]
Trypethelonamide A (**86**)	399	C_23_H_29_NO_5_	*Trypethelium eluteriae* Sprengl	-	Hainan, China	[52]
5′-Hydroxytrypethelone (**87**)	288	C_16_H_16_O_5_	*Trypethelium eluteriae* Sprengl	-	Hainan, China	[52]
(+)-8-Hydroxy-7-methoxytrypethelone (**88**)	302	C_17_H_18_O_5_	*Trypethelium eluteriae* Sprengl	-	Hainan, China	[52]
(+)-Trypethelone (**89**)	272	C_16_H_16_O_4_	*Trypethelium eluteriae* Sprengl	-	Hainan, China	[52]
			*Trypethelium eluteriae* Sprengl	*Anacardium occidentale* (Bark, Anacardiaceae)	Vadanemmeli, Kanchipuram, Tamil Nadu, India	[41]
(−)-Trypethelone (**90**)	272	C_16_H_16_O_4_	*Trypethelium eluteriae* Sprengl	-	Hainan, China	[52]
			*Pseudolophiostoma* sp. MFLUCC 17-2081	*Clematis fulvicoma* (Ranunculaceae)	Chiang Rai, Thailand	[45]
			*Coniothyrium cereale*	*Enteromorpha* sp. (Algae, Ulvaceae)	Fehmarn, Baltic Sea, Germany	[20]
Trypethelone methyl ether (**91**)	286	C_17_H_18_O_4_	*Trypethelium eluteriae* Sprengl	*Anacardium occidentale* (Bark, Anacardiaceae)	Vadanemmeli, Kanchipuram, Tamil Nadu, India	[41]
8-Hydroxytrypethelone methyl ether (**92**)	302	C_17_H_18_O_5_	*Trypethelium eluteriae* Sprengl	*Anacardium occidentale* (Bark, Anacardiaceae)	Vadanemmeli, Kanchipuram, Tamil Nadu, India	[41]
8-Methoxytrypethelone (**93**)	302	C_17_H_18_O_5_	*Trypethelium eluteriae* Sprengl	*Anacardium occidentale* (Bark, Anacardiaceae)	Vadanemmeli, Kanchipuram, Tamil Nadu, India	[41]
4′-Hydroxy-8-methoxytrypethelone methyl ether (**94**)	332	C_18_H_20_O_6_	*Trypethelium eluteriae* Sprengl	*Anacardium occidentale* (Bark, Anacardiaceae)	Vadanemmeli, Kanchipuram, Tamil Nadu, India	[41]
5′-Hydroxy-8-ethoxytrypethelone (**95**)	332	C_18_H_20_O_6_	*Trypethelium eluteriae* Sprengl	*Anacardium occidentale* (Bark, Anacardiaceae)	Vadanemmeli, Kanchipuram, Tamil Nadu, India	[41]
8-Methoxytrypethelone methyl ether (**96**)	316	C_18_H_20_O_5_	*Trypethelium eluteriae* Sprengl	*Anacardium occidentale* (Bark, Anacardiaceae)	Vadanemmeli, Kanchipuram, Tamil Nadu, India	[41]
Isoconiolactone (**97**)	300	C_17_H_16_O_5_	*Chrysosporium lobatum* TM-237-S5	*Acanthella cavernosa* (Sponge, Dictyonellidae)	Mesophotic reef in Eilat, Dekel Beach, Gulf of Aqaba, Israel	[40]
Coniolactone (**98**)	300	C_17_H_16_O_5_	*Chrysosporium lobatum* TM-237-S5	*Acanthella cavernosa* (Sponge, Dictyonellidae)	Mesophotic reef in Eilat, Dekel Beach, Gulf of Aqaba, Israel	[40]
			*Coniothyrium cereale*	*Enteromorpha* sp. (Algae, Ulvaceae)	Fehmarn, Baltic Sea, Germany	[20]
Coniosclerodin (**99**)	328	C_18_H_16_O_6_	*Chrysosporium lobatum* TM-237-S5	*Acanthella cavernosa* (Sponge, Dictyonellidae)	Mesophotic reef in Eilat, Dekel Beach, Gulf of Aqaba, Israel	[40]
			*Coniothyrium cereale*	*Enteromorpha* sp. (Algae, Ulvaceae)	Fehmarn, Baltic Sea, Germany	[20]
*O*-Desmethylfunalenone (**100**)	274	C_14_H_10_O_6_	*Talaromyces stipitatus*	Soil	Collaroy, New South Wales, Australia	[49]
Funalenone (**101**)	288	C_15_H_12_O_6_	*Aspergillus* sp. SF-5929.	Coral	Ross Sea, Antarctica	[53]
Hispidulone A (**102**)	276	C_14_H_12_O_6_	*Chaetosphaeronema hispidulum* TS-8-1	Desert plant	Desert, arid, and grassland areas, China	[54]
Hispidulone B (**103**)	290	C_15_H_14_O_6_	*Chaetosphaeronema hispidulum* TS-8-1	Desert plant	Desert, arid, and grassland areas, China	[54]
Aceneoherqueinone A (**104**)	414	C_22_H_22_O_8_	*Penicillium herquei* MA-370	Rhizospheric soil of the mangrove plant *Rhizophora mucronata* (Rhizophoraceae)	Hainan Island, China	[46]
Aceneoherqueinone B (**105**)	414	C_22_H_22_O_8_	*Penicillium herquei* MA-370	Rhizospheric soil of the mangrove plant *Rhizophora mucronata* (Rhizophoraceae)	Hainan Island, China	[46]
(+)-Aceatrovenetinone A (**106**)	398	C_22_H_22_O_7_	*Penicillium herquei* MA-370	Rhizospheric soil of the mangrove plant *Rhizophora mucronata* (Rhizophoraceae)	Hainan Island, China	[46]
(−)-Aceatrovenetinone B (**107**)	398	C_22_H_22_O_7_	*Penicillium herquei* MA-370	Rhizospheric soil of the mangrove plant *Rhizophora mucronata* (Rhizophoraceae)	Hainan Island, China	[46]
(−)-aceatrovenetinone A (**108**)	398	C_22_H_22_O_7_	*Penicillium herquei* MA-370	Rhizospheric soil of the mangrove plant *Rhizophora mucronata* (Rhizophoraceae)	Hainan Island, China	[46]
(+)-Aceatrovenetinone B (**109**)	398	C_22_H_22_O_7_	*Penicillium herquei* MA-370	Rhizospheric soil of the mangrove plant *Rhizophora mucronata* (Rhizophoraceae)	Hainan Island, China	[46]
Lamellicolic anhydride (**110**)	260.	C_13_H_8_O_6_	*Coniothyrium cereale*	*Enteromorpha* sp. (Algae, Ulvaceae)	Fehmarn, Baltic Sea, Germany	[20]
(−)-Cereoaldomine (**111**)	301	C_16_H_15_NO_5_	*Coniothyrium cereale*	*Enteromorpha* sp. (Algae, Ulvaceae)	Fehmarn, Baltic Sea, Germany	[20]
Z-Coniosclerodinol (**112**)	344	C_18_H_16_O_7_	*Coniothyrium cereale*	*Enteromorpha* sp. (Algae, Ulvaceae)	Fehmarn, Baltic Sea, Germany	[20]
E-Coniosclerodinol (**113**)	344	C_18_H_16_O_7_	*Coniothyrium cereale*	*Enteromorpha* sp. (Algae, Ulvaceae)	Fehmarn, Baltic Sea, Germany	[20]
Conioscleroderolide (**114**)	328	C_18_H_16_O_6_	*Coniothyrium cereale*	*Enteromorpha* sp. (Algae, Ulvaceae)	Fehmarn, Baltic Sea, Germany	[20]
Cereolactam (**115**)	299	C_17_H_17_NO_4_	*Coniothyrium cereale*	*Enteromorpha* sp. (Algae, Ulvaceae)	Fehmarn, Baltic Sea, Germany	[20]
Herqueilenone A (**116**)	581	C_30_H_31_NO_11_	*Penicillium herquei* FT729	Volcanic soil sample	Big Island, Hawaii	[55]
Erabulenol B (**117**)	550	C_30_H_30_O_10_	*Penicillium herquei* FT729	Volcanic soil sample	Big Island, Hawaii	[55]
Erabulenol C (**118**)	550	C_30_H_30_O_10_	*Penicillium herquei* FT729	Volcanic soil	Big Island, Hawaii	[55]
Duclauxamide A1 (**119**)	557	C_30_H_23_NO_10_	*Penicillium manginii* YIM PH30375	*Panax notoginseng* (Root, Araliaceae)	Wenshan, Yunnan, China	[56]
Duclauxin (**120**)	546	C_29_H_22_O_11_	*Penicillium manginii* YIM PH30375	*Panax notoginseng* (Root, Araliaceae)	Wenshan, Yunnan, China	[56]
			*Talaromyces verruculosus*	*Goniopora* sp. (Soft coral, Poritidae)	Sanya, Hainan Island, South China Sea, China	[57]
			*Talaromyces stipitatus*	Soil	Collaroy, New South Wales, Australia	[49]
			*Talaromyces* sp. IQ-313	Anthill soil	Huasteca Hidalguense, Hidalgo State, Mexico	[58]
Talaromycesone A (**121**)	548	C_29_H_24_O_11_	*Talaromyces* sp. LF458	*Axinellaverrucosa* (Sponge, Axinellidae)	Punta di Fetovaia, Isle of Elba, Mediterranean Sea, Italy	[59]
Talaromycesone B (**122**)	502	C_27_H_18_O_10_	*Talaromyces* sp. LF458	*Axinellaverrucosa* (Sponge, Axinellidae)	Punta di Fetovaia, Isle of Elba, Mediterranean Sea, Italy	[59]
			*Talaromyces* sp. IQ-313	Anthill soil	Huasteca Hidalguense, Hidalgo State, Mexico.	[58]
Bacillisporin A (**123**)	516	C_28_H_20_O_10_	*Talaromyces stipitatus* ATCC 10500	Culture	France	[60]
9a-*Epi*-bacillisporin E (**124**)	532	C_28_H_20_O_11_	*Talaromyces stipitatus* ATCC 10500	Culture	France	[60]
Bacillisporin F (**125**)	546	C_29_H_22_O_11_	*Talaromyces stipitatus* ATCC 10500	Culture	France	[60]
			*Talaromyces verruculosus*	*Goniopora* sp. (Soft coral, Poritidae)	Sanya, Hainan Island, South China Sea, China	[57]
1- *Epi*-bacillisporin F (**126**)	546	C_29_H_22_O_11_	*Talaromyces stipitatus* ATCC 10500	Culture	France	[60]
Bacillisporin G (**127**)	488	C_27_H_20_O_9_	*Talaromyces stipitatus* ATCC 10500	Culture	France	[60]
			*Talaromyces* sp. IQ-313	Anthill soil	Huasteca Hidalguense, Hidalgo State, Mexico.	[58]
Bacillisporin H (**128**)	545	C_29_H_23_NO_10_	*Talaromyces stipitatus* ATCC 10500	Culture	France	[60]
Verruculosin A (**129**)	604	C_32_H_28_O_12_	*Talaromyces verruculosus*	*Goniopora* sp. (Soft coral, Poritidae)	Sanya, Hainan Island, South China Sea, China	[57]
Verruculosin B (**130**)	620	C_32_H_28_O_13_	*Talaromyces verruculosus*	*Goniopora* sp. (Soft coral, Poritidae)	Sanya, Hainan Island, South China Sea, China	[57]
Xenoclauxin (**131**)	562	C_29_H_22_O_12_	*Talaromyces verruculosus*	*Goniopora* sp. (Soft coral, Poritidae)	Sanya, Hainan Island, South China Sea, China	[57]
			*Talaromyces* sp. IQ-313	Anthill soil	Huasteca Hidalguense, Hidalgo State, Mexico.	[58]
Talauxin E (**132**)	675	C_34_H_29_NO_14_	*Talaromyces stipitatus*	Soil	Collaroy, New South Wales, Australia	[49]
Talauxin I (**133**)	659	C_35_H_33_NO_12_	*Talaromyces stipitatus*	Soil	Collaroy, New South Wales, Australia	[49]
Talauxin L (**134**)	659	C_35_H_33_NO_12_	*Talaromyces stipitatus*	Soil	Collaroy, New South Wales, Australia	[49]
Talauxin Q (**135**)	674	C_34_H_30_N_2_O_13_	*Talaromyces stipitatus*	Soil	Collaroy, New South Wales, Australia	[49]
Talauxin V (**136**)	645	C_34_H_31_NO_12_	*Talaromyces stipitatus*	Soil	Collaroy, New South Wales, Australia	[49]
Epitalauxin I (**137**)	659	C_35_H_33_NO_12_	*Talaromyces stipitatus*	Soil	Collaroy, New South Wales, Australia	[49]
Epitalauxin L (**138**)	659	C_35_H_33_NO_12_	*Talaromyces stipitatus*	Soil	Collaroy, New South Wales, Australia	[49]
			*Pseudolophiostoma* sp. MFLUCC 17-2081	*Clematis fulvicoma* (Ranunculaceae)	Chiang Rai, Thailand	[45]
Desacetyldesmethyltalauxin V (**139**)	571	C_31_H_25_NO_10_	*Talaromyces stipitatus*	Soil sample	Collaroy, New South Wales, Australia	[49]

**Table 2:** Biological activities of the most active fungal phenalenones.

Compound Name	Biological Activity	Assay, Organism, or Cell Line	Biological Results	Positive Control	Ref.
Paecilomycone A (**1**)	Tyrosinase inhibition	Colorimetric-microtiter plates/Tyrosinase enzyme	0.11 mM (IC_50_)	Kojic acid 0.10 mM (IC_50_) Arbutin 0.20 mM (IC_50_)	[32]
Paecilomycone B (**2**)	Tyrosinase inhibition	Colorimetric-microtiter plates/Tyrosinase enzyme	0.17 mM (IC_50_)	Kojic acid 0.10 mM (IC_50_) Arbutin 0.20 mM (IC_50_)	[32]
Paecilomycone C (**3**)	Tyrosinase inhibition	Colorimetric-microtiter plates/Tyrosinase enzyme	0.14 mM (IC_50_)	Kojic acid 0.10 mM (IC_50_) Arbutin 0.20 mM (IC_50_)	[32]
Aspergillussanone A (**5**)	Cytotoxicity	Resazurin microplate/KB	48.4 μM (IC_50_)	Ellipticine 4.1 μM (IC_50_)	[33]
		Resazurin microplate/Vero	34.2 μM (IC_50_)	Ellipticine 4.5 μM (IC_50_)	[33]
*ent*-Peniciherqueinone (**8**)	Adipogenesis induction	Adiponectin production assay/hBM-MSC(B7)	57.5 µM (IC_50_)	Pioglitazone 0.69 µM (IC_50_)	[35]
Herqueinone (**9**)	Antioxidant	DPPH/DPPH^•^	0.48 mM (IC_50_)	Butylated hydroxytoluene 0.11 mM (IC_50_)	[34]
		Hydroxyl radical scavenging/OH^•^	6.34 mM (IC_50_)	Tannic acid 0.26 mM (IC_50_)	[34]
		Superoxide radical scavenging/O_2_^•−^	4.11 mM (IC_50_)	Trolox 0.96 mM (IC_50_)	[34]
Isoherqueinone (**12**)	Adipogenesis induction	Adiponectin production assay/hBM-MSC(B7)	39.7 µM (IC_50_)	Pioglitazone 0.69 µM (IC_50_)	[35]
Acetone adduct of a triketone (**17**)	Anti-inflammatory	Nitric oxide synthase/RAW 264.7	3.2 µM (IC_50_)	AMT 0.2 µM (IC_50_)	[35]
(+)-Sclerodin (**21**)	Cytotoxicity	SRB/U87MG	55.99 µM (IC_50_)	Doxorubicin 1.2 µM (IC_50_)	[39]
		SRB/C6	44.65 µM (IC_50_)	Doxorubicin 0.47 µM (IC_50_)	[39]
(−)-Sclerodinol (**24**)	Antimicrobial	Agar dilution/*B. cereus*	25.0 µM (MIC)	Ciprofloxacin 0.39 µM (MIC)	[44]
		Agar dilution/*Proteus* species	50.0 µM (MIC)	Ciprofloxacin 0.78 µM (MIC)	[44]
		Agar dilution/*M. Phlei*	25.0 µM (MIC)	Ciprofloxacin 0.39 µM (MIC)	[44]
		Agar dilution/*B. subtilis*	12.5 µM (MIC)	Ciprofloxacin 0.39 µM (MIC)	[44]
		Agar dilution/*V. parahemolyticus*	12.5 µM (MIC)	Ciprofloxacin 0.39 µM (MIC)	[44]
		Agar dilution/MRCNS	50.0 µM (MIC)	Ciprofloxacin 25.0 µM (MIC)	[44]
		Agar dilution/MRSA	50.0 µM (MIC)	Ciprofloxacin > 50 µM (MIC)	[44]
Bipolarol C (**26**)	Antibacterial	REMA/*B. cereus*	25.0 µg/mL (MIC)	Vancomycin 1.0 µg/mL (MIC)	[42]
4-Hydroxysclerodin (**27**)	Anti-angiogenetic	Tube formation assay/HUVECs	20.9 µM (IC_50_)	Sunitinib 1.5 µM (IC_50_)	[35]
(+)-Sclerodione (**28**)	Cytotoxicity	SRB/U87MG	60.93 µM (IC_50_)	Doxorubicin 1.2 µM (IC_50_)	[39]
		SRB/C6	60.81 µM (IC_50_)	Doxorubicin 0.47 µM (IC_50_)	[39]
	Antibacterial	Micro-broth dilution/MRSA	23.0 µg/mL (MIC)	Gentamicin 0.5 µg/mL (MIC)	[39]
		Micro-broth dilution/*E*. *coli*	35.0 µg/mL (MIC)	Gentamicin 1.0 µg/mL (MIC)	[39]
(−)–Sclerodione (**29**)	α-Glucosidase inhibition	Colorimetric/α-Glucosidase	120 µM (IC_50_)	N-deoxynojirimycin 130.5 µM (IC_50_)	[45]
	Porcine-lipase inhibition	Colorimetric/Porcine lipase	1.0 µM (IC_50_)	Orlistat 9.4 µM (IC_50_)	[45]
(−)-Bipolaride B (**30**)	Antibacterial	REMA/*B. cereus*	25.0 µg/mL (MIC)	Vancomycin 1.0 µg/mL (MIC)	[42]
	Cytotoxicity	REMA/MCF-7	79.4 µM (IC_50_)	Doxorubicin 12.99 µM (IC_50_) Tamoxifen 19.39 µM (IC_50_)	[42]
		REMA/KB	96.8 µM (IC_50_)	Ellipticine 8.32 µM (IC_50_) Doxorubicin 1.15 µM (IC_50_)	[42]
		REMA/NCI-H187	56.5 µM (IC_50_)	Ellipticine 9.74 µM (IC_50_) Doxorubicin 0.19 µM (IC_50_)	[42]
Peniciphenalenin H (**31**)	Antimicrobial	Agar dilution/*Proteus* species	50.0 µM (MIC)	Ciprofloxacin 0.78 µM (MIC)	[44]
		Agar dilution/*B. subtilis*	25.0 µM (MIC)	Ciprofloxacin 0.39 µM (MIC)	[44]
		Agar dilution/*V. parahemolyticus*	25.0 µM (MIC)	Ciprofloxacin 0.39 µM (MIC)	[44]
		Agar dilution/MRCNS	25.0 µM (MIC)	Ciprofloxacin 25.0 µM (MIC)	[44]
		Agar dilution/MRSA	50.0 µM (MIC)	Ciprofloxacin > 50 µM (MIC)	[44]
Bipolarol A (**32**)	Cytotoxicity	REMA/MCF-7	110.4 µM (IC_50_)	Doxorubicin 12.99 µM (IC_50_) Tamoxifen 19.39 µM (IC_50_)	[42]
(+)-Scleroderolide (**33**)	Cytotoxicity	SRB/U87MG	37.26 µM (IC_50_)	Doxorubicin 1.2 µM (IC_50_)	[39]
		SRB/C6	23.24 µM (IC_50_)	Doxorubicin 0.47 µM (IC_50_)	[39]
	Antibacterial	Micro-broth dilution/MRSA	7.0 µg/mL (MIC)	Gentamicin 0.5 µg/mL (MIC)	[39]
		Micro-broth dilution/*E*. *coli*	9.0 µg/mL (MIC)	Gentamicin 1.0 µg/mL (MIC)	[39]
(−)–Scleroderolide (**34**)	α-Glucosidase inhibition	Colorimetric/α-Glucosidase	48.7 µM (IC_50_)	*N*-Deoxynojirimycin 130.5 µM (IC_50_)	[45]
	porcine-lipase inhibition	Colorimetric/Porcine lipase	3.4 µM (IC_50_)	Orlistat 9.4 µM (IC_50_)	[45]
(−)-Bipolaride A (**36**)	Antibacterial	REMA/*B. cereus*	12.5 µg/mL (MIC)	Vancomycin 1.0 µg/mL (MIC)	[42]
	Cytotoxicity	REMA/NCI-H187	60.2 µM (IC_50_)	Ellipticine 9.74 µM (IC_50_) Doxorubicin 0.19 µM (IC_50_)	[42]
(−)-Bipolaride E (**39**)	Antibacterial	REMA/*B. cereus*	12.5 µg/mL (MIC)	Vancomycin 1.0 µg/mL (MIC)	[42]
	Cytotoxicity	REMA/MCF-7	65.1 µM (IC_50_)	Doxorubicin 12.99 µM (IC_50_) Tamoxifen 19.39 µM (IC_50_)	[42]
		REMA/KB	94.4 µM (IC_50_)	Ellipticine 8.32 µM (IC_50_) Doxorubicin 1.15 µM (IC_50_)	[42]
		REMA/NCI-H187	86.9 µM (IC_50_)	Ellipticine 9.74 µM (IC_50_) Doxorubicin 0.19 µM (IC_50_)	[42]
Bipolarol B (**40**)	Cytotoxicity	REMA/MCF-7	65.3 µM (IC_50_)	Doxorubicin 12.99 µM (IC_50_) Tamoxifen 19.39 µM (IC_50_)	[42]
		REMA/KB	52.5 µM (IC_50_)	Ellipticine 8.32 µM (IC_50_) Doxorubicin 1.15 µM (IC_50_)	[42]
		REMA/NCI-H187	48.3 µM (IC_50_)	Ellipticine 9.74 µM (IC_50_) Doxorubicin 0.19 µM (IC_50_)	[42]
Bipolarol D (**41**)	REMA/MCF-7	REMA/MCF-7	108.7 µM (IC_50_)	Doxorubicin 12.99 µM (IC_50_) Tamoxifen 19.39 µM (IC_50_)	[42]
(−)-Bipolaride C (**42**)	Antibacterial	REMA/*B. cereus*	12.5 µg/mL (MIC)	Vancomycin 1.0 µg/mL (MIC)	[42]
	Cytotoxicity	REMA/MCF-7	48.9 µM (IC_50_)	Doxorubicin 12.99 µM (IC_50_) Tamoxifen 19.39 µM (IC_50_)	[42]
		REMA/KB	34.4 µM (IC_50_)	Ellipticine 8.32 µM (IC_50_) Doxorubicin 1.15 µM (IC_50_)	[42]
		REMA/NCI-H187	59.8 µM (IC_50_)	Ellipticine 9.74 µM (IC_50_) Doxorubicin 0.19 µM (IC_50_)	[42]
		REMA/Vero	53.1 µM (IC_50_)	Ellipticine 2.13 µM (IC_50_)	[42]
Flaviphenalenone A (**45**)	Cytotoxicity	MTT/A549	6.6 µg/mL (IC_50_)	Doxorubicin HCl 0.2 µg/mL (IC_50_)	[31]
		MTT/MCF-7	10.0 µg/mL (IC_50_)	Doxorubicin HCl 0.4 µg/mL (IC_50_)	[31]
Flaviphenalenone B (**46**)	α-Glucosidase inhibition	Colorimetrically/α-Glucosidase	94.95 µM (IC_50_)	Acarbose 685.36 µM (IC_50_)	[31]
Flaviphenalenone C (**47**)	α-Glucosidase inhibition	Colorimetrically/α-Glucosidase	78.96 µM (IC_50_)	Acarbose 685.36 µM (IC_50_)	[31]
	Cytotoxicity	MTT/A549	28.5 µg/mL (IC_50_)	Doxorubicin HCl 0.2 µg/mL (IC_50_)	[31]
		MTT/MCF-7	50.0 µg/mL (IC_50_)	Doxorubicin HCl 0.4 µg/mL (IC_50_)	[31]
Auxarthrone A (**49**)	Antifungal	Serial dilution/*C. neoformans*	3.2 µg/mL (MIC)	Amphotericin B 0.8 µg/mL (MIC)	[48]
		Serial dilution/*C. albicans*	3.2 µg/mL (MIC)	Amphotericin B 0.8 µg/mL (MIC)	[48]
Auxarthrone B (**50**)	Antifungal	Serial dilution/*C. neoformans*	12.8 µg/mL (MIC)	Amphotericin B 0.8 µg/mL (MIC)	[48]
		Serial dilution/*C. albicans*	25.6 µg/mL (MIC)	Amphotericin B 0.8 µg/mL (MIC)	[48]
Auxarthrone D (**51**)	Antifungal	Serial dilution/*C. neoformans*	6.4 µg/mL (MIC)	Amphotericin B 0.8 µg/mL (MIC)	[48]
		Serial dilution/*C. albicans*	6.4 µg/mL (MIC)	Amphotericin B 0.8 µg/mL (MIC)	[48]
Auxarthrone C (**52**)	Antifungal	Serial dilution/*C. neoformans*	25.6 µg/mL (MIC)	Amphotericin B 0.8 µg/mL (MIC)	[48]
		Serial dilution/*C. albicans*	51.2 µg/mL (MIC)	Amphotericin B 0.8 µg/mL (MIC)	[48]
FR-901235 (**54**)	Antifungal	Serial dilution/*C. neoformans*	51.2 µg/mL (MIC)	Amphotericin B 0.8 µg/mL (MIC)	[48]
		Serial dilution/*C. albicans*	51.2 µg/mL (MIC)	Amphotericin B 0.8 µg/mL (MIC)	[48]
Aspergillussanone D (**61**)	Antibacterial	Broth microdilution/*P. aeruginosa*	38.47 µg/mL (MIC_50_)	Streptomycin 0.34 µg/mL (MIC_50_)	[50]
		Broth microdilution/*S. aureus*	29.91 µg/mL (MIC_50_)	Penicillin 0.063 µg/mL (MIC_50_)	[50]
Aspergillussanone E (**62**)	Antibacterial	Broth microdilution/*E. coli*	7.83 µg/mL (MIC_50_)	Streptomycin 0.34 µg/mL (MIC_50_)	[50]
Aspergillussanone F (**63**)	Antibacterial	Broth microdilution/*P. aeruginosa*	26.56 µg/mL (MIC_50_)	Streptomycin 0.34 µg/mL (MIC_50_)	[50]
		Broth microdilution/*E. coli*	3.93 µg/mL (MIC_50_)	Streptomycin 0.25 µg/mL (MIC_50_)	[50]
		Broth microdilution/*S. aureus*	16.48 µg/mL (MIC_50_)	Penicillin 0.063 µg/mL (MIC_50_)	[50]
Aspergillussanone G (**64**)	Antibacterial	Broth microdilution/*P. aeruginosa*	24.46 µg/mL (MIC_50_)	Streptomycin 0.34 µg/mL (MIC_50_)	[50]
		Broth microdilution/*S. aureus*	34.66 µg/mL (MIC_50_)	Penicillin 0.063 µg/mL (MIC_50_)	[50]
Aspergillussanone H (**65**)	Antibacterial	Broth microdilution/*P. aeruginosa*	8.59 µg/mL (MIC_50_)	Streptomycin 0.34 µg/mL (MIC_50_)	[50]
		Broth microdilution/*E. coli*	5.87 µg/mL (MIC_50_)	Streptomycin 0.25 µg/mL (MIC_50_)	[50]
Aspergillussanone I (**66**)	Antibacterial	Broth microdilution/*P. aeruginosa*	12.00 µg/mL (MIC_50_)	Streptomycin 0.34 µg/mL (MIC_50_)	[50]
Aspergillussanone J (**67**)	Antibacterial	Broth microdilution/*P. aeruginosa*	28.50 µg/mL (MIC_50_)	Streptomycin 0.34 µg/mL (MIC_50_)	[50]
		Broth microdilution/*E. coli*	5.34 µg/mL (MIC_50_)	Streptomycin 0.25 µg/mL (MIC_50_)	[50]
		Broth microdilution/*S. aureus*	29.87 µg/mL (MIC_50_)	Penicillin 0.063 µg/mL (MIC_50_)	[50]
Aspergillussanone K (**68**)	Antibacterial	Broth microdilution/*P. aeruginosa*	6.55 µg/mL (MIC_50_)	Streptomycin 0.34 µg/mL (MIC_50_)	[50]
		Broth microdilution/*S. aureus*	21.02 µg/mL (MIC_50_)	Penicillin 0.063 µg/mL (MIC_50_)	[50]
Aspergillussanone L (**69**)	Antibacterial	Broth microdilution/*P. aeruginosa*	1.87 µg/mL (MIC_50_)	Streptomycin 0.34 µg/mL (MIC_50_)	[50]
		Broth microdilution/*S. aureus*	2.77 µg/mL (MIC_50_)	Penicillin 0.063 µg/mL (MIC_50_)	[50]
		Broth microdilution/*B. subtilis*	4.80 µg/mL (MIC_50_)	Penicillin 0.063 µg/mL (MIC_50_)	[50]
(*S*)-2-((*S,*2*E,*6*E,*10*Z*)-14,15-Dihydroxy-11-(hydroxymethyl)-3,7,15-trimethylhexadeca-2,6,10-trien-1-yl)-2,4,6,9-tetrahydroxy-5,7-dimethyl-1*H*-phenalene-1,3(2*H*)-dione (**70**)	Antibacterial	Broth microdilution/*P. aeruginosa*	19.07 µg/mL (MIC_50_)	Streptomycin 0.34 µg/mL (MIC_50_)	[50]
		Broth microdilution/*E. coli*	1.88 µg/mL (MIC_50_)	Streptomycin 0.25 µg/mL (MIC_50_)	[50]
Asperphenalenone A (**71**)	Anti-HIV-1	Luciferase assay/SupT1 cells	4.2 µM (IC_50_)	Lamivudine 0.1 µM (IC_50_) Efavirenz 0.0004 µM (IC_50_)	[51]
Asperphenalenone B (**72**)	Anti-HIV-1	Luciferase assay/SupT1 cells	32.6 µM (IC_50_)	Lamivudine 0.1 µM (IC_50_) Efavirenz 0.0004 µM (IC_50_)	[51]
Asperphenalenone D (**74**)	Anti-HIV-1	Luciferase assay/SupT1 cells	2.4 µM (IC_50_)	Lamivudine 0.1 µM (IC_50_) Efavirenz 0.0004 µM (IC_50_)	[51]
	Anti-HIV-1	Luciferase assay/SupT1 cells	22.1 µM (IC_50_)	Lamivudine 0.1 µM (IC_50_) Efavirenz 0.0004 µM (IC_50_)	[51]
Peniciphenalenin G (**83**)	Antimicrobial	Agar dilution/*B. cereus*	25.0 µM (MIC)	Ciprofloxacin 0.39 µM (MIC)	[44]
		Agar dilution/*Proteus* species	50.0 µM (MIC)	Ciprofloxacin 0.78 µM (MIC)	[44]
		Agar dilution/*M. Phlei*	50.0 µM (MIC)	Ciprofloxacin 0.39 µM (MIC)	[44]
		Agar dilution/*B. subtilis*	25.0 µM (MIC)	Ciprofloxacin 0.39 µM (MIC)	[44]
		Agar dilution/*V. Parahemolyticus*	50.0 µM (MIC)	Ciprofloxacin 0.39 µM (MIC)	[44]
		Agar dilution/MRCNS	25.0 µM (MIC)	Ciprofloxacin 25.0 µM (MIC)	[44]
		Agar dilution/MRSA	12.5 µM (MIC)	Ciprofloxacin > 50 µM (MIC)	[44]
Coniosclerodione (**85**)	Antimicrobial	Agar dilution/*B. cereus*	25.0 µM (MIC)	Ciprofloxacin 0.39 µM (MIC)	[44]
		Agar dilution/*Proteus* sp.	25.0 µM (MIC)	Ciprofloxacin 0.78 µM (MIC)	[44]
		Agar dilution/*M. Phlei*	25.0 µM (MIC)	Ciprofloxacin 0.39 µM (MIC)	[44]
		Agar dilution/*B. subtilis*	12.5 µM (MIC)	Ciprofloxacin 0.39 µM (MIC)	[44]
		Agar dilution/*V. parahemolyticus*	12.5 µM (MIC)	Ciprofloxacin 0.39 µM (MIC)	[44]
		Agar dilution/MRCNS	12.5 µM (MIC)	Ciprofloxacin 25.0 µM (MIC)	[44]
		Agar dilution/MRSA	6.25 µM (MIC)	Ciprofloxacin > 50 µM (MIC)	[44]
Trypethelonamide A (**86**)	Cytotoxicity	CCK8/RKO	63.6 µM (IC_50_)	Taxol 0.05 µM (IC_50_)	[52]
5′-Hydroxytrypethelone (**87**)	Cytotoxicity	CCK8/RKO	22.6 µM (IC_50_)	Taxol 0.05 µM (IC_50_)	[52]
(+)-8-Hydroxy-7-methoxytrypethelone (**88**)	Cytotoxicity	CCK8/RKO	113.5 µM (IC_50_)	Taxol 0.05 µM (IC_50_)	[52]
		CCK8/HepG2	183.2 µM (IC_50_)	Taxol 1.0 µM (IC_50_)	[52]
(+)-Trypethelone (**89**)	Cytotoxicity	CCK8/RKO	49.3 µM (IC_50_)	Taxol 0.05 µM (IC_50_)	[52]
(−)-Trypethelone (**90**)	Cytotoxicity	CCK8/RKO	30.3 µM (IC_50_)	Taxol 0.05 µM (IC_50_)	[52]
*O*-Desmethylfunalenone (**100**)	Antibacterial	REMA/*B. subtilis*	265 µM (IC_50_)	Clotrimazole 0.4 µM (IC_50_)	[49]
	Cytotoxicity	Resazurin microplate/NS-1	70 µM (IC_50_)	5-Fluorouracil 4.6 µM (IC_50_)	[49]
Funalenone (**101**)	*h*PTP1B_1-400_ inhibition	Photocolorimetric/*h*PTP1B_1-400_	6.1 µM (IC_50_)	Ursolic acid 4.3 µM (IC_50_)	[53]
Hispidulone B (**103**)	Cytotoxicity	MTT/A-549	2.71 µM (IC_50_)	*cis*-Platinum 8.73 µM (IC_50_)	[54]
		MTT/Huh7	22.93 µM (IC_50_)	*cis*-Platinum 5.89 µM (IC_50_)	[54]
		MTT/HeLa	23.94 µM (IC_50_)	*cis*-Platinum 14.68 µM (IC_50_)	[54]
Aceneoherqueinone A (**104**)	Angiotensin-I-converting enzyme inhibition	Spectrophotometric/Hippuryl-L-histidyl-L-leucine	3.10 µM (IC_50_)	Captopril 9.23 nM (IC_50_)	[46]
Aceneoherqueinone B (**105**)	Angiotensin-I-converting enzyme inhibition	Spectrophotometric/Hippuryl-L-histidyl-L-leucine	11.28 µM (IC_50_)	Captopril 9.23 nM (IC_50_)	[46]
Erabulenol B (**117**)	Indoleamine dioxygenase 1 inhibition	ELISA/Indoleamine 2,3-dioxygenase 1	13.69 µM (IC_50_)	Epacadostat 0.015 µM (IC_50_)	[55]
Erabulenol C (**118**)	Indoleamine dioxygenase 1 inhibition	ELISA/Indoleamine 2,3-dioxygenase 1	14.38 µM (IC_50_)	Epacadostat 0.015 µM (IC_50_)	[55]
Duclauxin (**120**)	Antitumor	ELISA/EGFR	0.95 µM (IC_50_)	Afatinib 0.0005 µM (IC_50_)	[57]
		ELISA/CDC25B	0.75 µM (IC_50_)	Na_3_VO_4_ 0.52 µM (IC_50_)	[57]
	Cytotoxicity	Resazurin microplate/NS-1	140 µM (IC_50_)	5-Fluorouracil 4.6 µM (IC_50_)	[49]
	*h*PTP1B_1-400_ inhibition	Photocolorimetric/*h*PTP1B_1-400_	12.7 µM (IC_50_)	Ursolic acid 26.6 µM (IC_50_)	[58]
Talaromycesone A (**121**)	Antibacterial	REMA/*S*. *epidermidis*	3.70 µM (IC_50_)	Chloramphenicol 1.81 µM (IC_50_)	[59]
	Antibacterial	REMA/MRSA	5.48 µM (IC_50_)	Chloramphenicol 2.46 µM (IC_50_)	[59]
	AchE inhibition	Modified Ellman’s enzyme/Immunosorbent assay	7.49 µM (IC_50_)	Huperzine 11.60 µM (IC_50_)	[59]
Talaromycesone B (**122**)	Antibacterial	REMA/*S*. *epidermidis*	17.36 µM (IC_50_)	Chloramphenicol 1.81 µM (IC_50_)	[59]
		REMA/MRSA	19.50 µM (IC_50_)	Chloramphenicol 2.46 µM (IC_50_)	[59]
	*h*PTP1B_1-400_ inhibition	Photocolorimetric/*h*PTP1B_1-400_	82.1 µM (IC_50_)	Ursolic acid 26.6 µM (IC_50_)	[58]
Bacillisporin A (**123**)	Antibacterial	Microtiter plate/*S*. *aureus*	5.2 µg/mL (MIC)	Tetracycline 0.05 µg/mL (MIC)	[60]
		Microtiter plate/*S*. *hemolyticus*	9.5 µg/mL (MIC)	Tetracycline 29.2 µg/mL (MIC)	[60]
		Microtiter plate/*E*. *faecalis*	2.4 µg/mL (MIC)	Tetracycline 0.4 µg/mL (MIC)	[60]
9a-*Epi*-bacillisporin E (**124**)	Antibacterial	Microtiter plate/*S*. *aureus*	29.3 µg/mL (MIC)	Tetracycline 0.05 µg/mL (MIC)	[60]
Bacillisporin F (**125**)	Antibacterial	Microtiter plate/*S*. *aureus*	15.6 µg/mL (MIC)	Tetracycline 0.05 µg/mL (MIC)	[60]
	Antitumor	ELISA/EGFR	4.41 µM (IC_50_)	Afatinib 0.0005 µM (IC_50_)	[57]
		ELISA/CDC25B	0.40 µM (IC_50_)	Na_3_VO_4_ 0.52 µM (IC_50_)	[57]
	Antitumor	ELISA/EGFR	4.41 µM (IC_50_)	Afatinib 0.0005 µM (IC_50_)	[57]
		ELISA/CDC25B	0.40 µM (IC_50_)	Na_3_VO_4_ 0.52 µM (IC_50_)	[57]
Bacillisporin G (**127**)	*h*PTP1B_1-400_ inhibition	Photocolorimetric/*h*PTP1B_1-400_	13.5 µM (IC_50_)	Ursolic acid 26.6 µM (IC_50_)	[58]
Bacillisporin H (**128**)	Cytotoxicity	MTT/HeLa	49.5 µM (IC_50_)	Cisplatin 10.6 µM (IC_50_)	[60]
	Antibacterial	Microtiter plate/*S*. *aureus*	5.0 µg/mL (MIC)	Tetracycline 0.05 µg/mL (MIC)	[60]
		Microtiter plate/*S*. *hemolyticus*	20.4 µg/mL (MIC)	Tetracycline 29.2 µg/mL (MIC)	[60]
Verruculosin A (**129**)	Antitumor	ELISA/EGFR	0.92 µM (IC_50_)	Afatinib 0.0005 µM (IC_50_)	[57]
	Antitumor	ELISA/CDC25B	0.38 µM (IC_50_)	Na_3_VO_4_ 0.52 µM (IC_50_)	[57]
Verruculosin B (**130**)	Antitumor	ELISA/EGFR	1.22 µM (IC_50_)	Afatinib 0.0005 µM (IC_50_)	[57]
Xenoclauxin (**131**)	Antitumor	ELISA/EGFR	0.24 µM (IC_50_)	Afatinib 0.0005 µM (IC_50_)	[57]
		ELISA/CDC25B	0.26 µM (IC_50_)	Na_3_VO_4_ 0.52 µM (IC_50_)	[57]
	*h*PTP1B_1-400_ inhibition	Photocolorimetric/*h*PTP1B_1-400_	21.8 µM (IC_50_)	Ursolic acid 26.6 µM (IC_50_)	[58]

**Table 3:** The prediction of target probability and model accuracy for phenalenone derivatives against GLUT1, using the SuperPred target prediction web server.

Compound #	Probability *	Model Accuracy **
**7**	63.54%	98.75%
**118**	78.92%	98.75%
**71**	64.66%	98.75%
**69**	68%	99%
**70**	66%	99%
**72**	67.3%	99%
**75**	66%	99%
**73**	67.3%	99%
**68**	78%	99%
**5**	68%	99%
**67**	71.95%	98.75%
**6**	75%	99%
**66**	NA	NA
**62**	53%	99%
**61**	53%	99%
**63**	72%	99%
**64**	76%	99%
**60**	52.61%	98.75%
**74**	66%	99%
**65**	74%	99%

* The probability of the test compound binding to a specific target, as determined by the respective target machine learning model. ** The 10-fold cross-validation score of the respective logistic regression model is presented, as the model performance varies between different targets.

**Table 4:** Predicted in silico ADME properties of the phenalenone derivatives.

Title	Mol_MW	# Stars	Dipole	SASA	HBD	HBA	QPlogPo/w	QPlogS	QPlogKhsa	# Metab	QPlogBB	%Human Oral Absorption	QPlogHERG	CNS	# RtvFG
Recommended range	(130–725)	(0.0–5.0)	(1–12.50)	(300–1000)	(0–6)	(2.0–20.0)	(−2–6.5)	(−6.5–0.5)	(−1.5–1.5)	(1–8)	(−3–1.2)	(<25% poor; >80% high)	concern below −5	(−2 inactive) (+2 active)	(0–2)
**7**	388.373	0	3.013	566.818	2	6.5	1.851	−3.559	0.097	6	−1.437	74.987	−3.478	−2	0
**118**	550.561	1	7.485	794.384	2	8.25	3.782	−6.151	0.715	9	−2.297	69.345	−5.022	−2	0
**71**	576.728	3	8.802	910.147	3	6.7	6.314	−7.258	1.316	18	−2.368	79.595	−5.491	−2	0
**69**	594.744	2	9.484	789.214	4	7.45	5.322	−4.622	0.981	17	−2.174	57.778	−3.532	−2	0
**70**	610.743	7	9.975	1043.134	5	9.15	5.066	−7.89	0.907	17	−4.262	41.015	−6.435	−2	0
**72**	624.727	8	8.459	1026.611	5	9.45	5.31	−7.68	0.735	16	−4.35	28.338	−4.482	−2	1
**75**	610.743	7	9.879	1030.596	5	9.15	4.911	−7.677	0.918	17	−4.46	49.169	−6.255	−2	0
**73**	624.727	8	8.459	1026.616	5	9.45	5.31	−7.68	0.735	16	−4.351	28.338	−4.482	−2	1
**68**	652.78	8	12.335	1096.494	4	9.45	6.114	−8.908	1.288	17	−4.061	50.421	−6.561	−2	1
**5**	540.652	3	14.173	889.502	3	7.45	5.255	−6.976	0.997	13	−2.485	70.591	−5.556	−2	0
**67**	666.807	8	10.067	1027.334	3	9.45	6.512	−7.869	1.359	16	−3.158	73.463	−5.735	−2	1
**6**	540.652	2	7.928	919.785	3	7.45	5.282	−7.502	1.064	13	−2.834	67.029	−5.832	−2	0
**66**	634.808	6	10.74	970.033	2	6.5	7.963	−8.898	1.935	15	−1.591	100	−5.533	−2	1
**62**	592.728	6	12.392	1005.377	3	7.45	6.358	−9.167	1.54	17	−2.88	73.816	−6.238	−2	0
**61**	592.728	6	11.434	1011.989	3	7.45	6.379	−9.284	1.561	17	−2.954	73.171	−6.288	−2	0
**63**	592.771	5	11.447	968.98	3	5.75	7.264	−8.949	1.821	13	−2.355	85.225	−5.777	−2	0
**64**	608.77	5	12.352	986.08	4	7.45	5.988	−8.302	1.398	14	−3.232	53.649	−5.838	−2	0
**60**	624.727	4	11.95	974.752	4	11.15	4.249	−7.089	0.71	14	−3.346	53.716	−5.828	−2	3
**74**	654.796	7	10.567	1030.237	3	10.85	5.564	−7.91	1.034	14	−3.042	68.666	−6.017	−2	1
**65**	606.711	5	9.825	992.894	3	8.75	5.513	−8.33	1.235	15	−3.349	62.369	−6.083	−2	1

**Table 5:** The in silico docking results of phenalenone derivatives with GLUT1 (PDB: **5EQG**).

Title	Docking Score	XP GScore	Glide Emodel	XP GScore
**60**	−15.082	−15.777	−112.586	−14.777
**64**	−14.829	−15.239	−101.024	−14.239
**66**	−13.511	−15.227	−88.992	−14.227
**71**	−13.348	−15.063	−96.987	−14.063
**68**	−14.276	−14.973	−130.242	−13.973
**61**	−14.073	−14.77	−93.445	−13.77
**70**	−14.248	−14.658	−98.707	−13.658
**65**	−12.689	−14.404	−96.631	−13.404
**69**	−12.733	−14.277	−85.473	−13.277
**75**	−13.343	−14.04	−108.477	−13.04
**74**	−12.284	−14.002	−99.09	−13.002
**63**	−13.061	−13.757	−104.496	−12.757
**72**	−11.996	−13.711	−78.259	−12.711
**67**	−12.07	−13.571	−105.73	−12.571
**5**	−12.01	−13.511	−94.603	−12.511
**6**	−13.162	−13.466	−88.095	−12.466
**73**	−11.712	−13.361	−96.264	−12.361
**62**	−12.586	−13.283	−98.251	−12.283
**7**	−11.849	−12.42	−78.277	−11.42
5RE	−11.206	−11.207	−88.062	−10.207
**118**	−9.901	−11.187	−84.952	−10.187

## Data Availability

Not applicable.

## Abbreviations

A-549	Human lung carcinoma
ACE	Angiotensin-I-converting enzyme
AchE	Acetylcholinesterase
AMT	2-Amino-5,6-dihydro-6-methyl-4H-1,3-thiazine hydrochloride
ASPC-1	Human pancreas adenocarcinoma ascites metastasis
C6	Rat glioma
CCK8	Cell counting kit-8
CD25	Interleukin-2 receptor alpha chain
CD69	Cluster of Differentiation 69
CDC25B	Cell division cycle 25b
CFSE	Carboxyfluorescein succinimidyl ester
CYP2E1	Cytochrome P450 2E1
DFT	Density functional theory
DPPH	1,1-Diphenyl-2-picrylhydrazyl
DPPH^•^	1,1-diphenyl-2-picrylhydrazyl radical
EGFR	Epidermal growth factor receptor
ELISA	Enzyme-linked immunosorbent assay
GFP	Green fluorescent protein
GFPMA	Green fluorescent protein microplate assay
GIAO	Gauge invariant atomic orbital
H69AR	Human lung carcinoma
hBM-MSCs	Human bone marrow-mesenchymal stem cells
HeLa	Human cervix carcinoma
HepG2	Human hepatocellular carcinoma
HL-60	Human myeloid leukemia
HPLC	High-performance liquid chromatography
hPTP1B1-400	Human PTP1B1-400
HRESIMS	High resolution electrospray ionization mass spectrometry
HSCCC	High-speed counter-current chromatography
Huh7	Human hepatoma
HUVECs	Human umbilical vascular endothelial cells
IC_50_	Half inhibition concentration
IDO1	Indoleamine 2,3-dioxygenase 1
IKKα	Kappab kinase alpha
IL-2	Interleukin 2
K562	Human chronic myelogenous leukemia
KB	Human oral carcinoma cell lines
L-02	Human normal liver
LPS	Lipopolysaccharides
MAPK	Mitogen-activated protein kinase
MCF-7	Human breast adenocarcinoma
MDA-MB-231	Human breast cancer
MIC	Minimum inhibitory concentration
MRCNS	Methicillin-resistant coagulase-negative staphylococci
MRSA	Methicillin-resistant S. aureus
MTT	(3-(4,5-Dimethylthiazol-2-yl)-2,5-diphenyltetrazolium bromide)
Na3VO4	Sodium orthovanadate
NCI-H187	Human small cell lung cancer
NF-κB	Nuclear factor kappa
NO	Nitric oxide
NS-1	Mouse myeloma
O_2_^•−^	Superoxide anion
OH^•^	Hydroxyl radical
PC-12	Rat a pheochromocytoma adrenal medulla
PMA/A23187	Phorbol 12-myistrate 13-acetate
PPARγ	Peroxisome proliferator-activated receptor gamma
PTKs	Protein tyrosine-kinases
PTPs	Protein tyrosine-phosphatases
REMA	Resazurin microplate assay
RKO	Human colorectal cancers
ROS	Reactive oxygen species
SKM1	Myelodysplastic symdrom
SMMC-7721	Hepatocellular carcinoma
SRB	Sulforhodamine B assay
SUP T1	Human T cell lymphoblastic
SW480	Hyman colon carcinoma
TAK1	Transforming growth factor beta-activated kinase 1
TCR	T cell receptor
U266	Myeloma
U87MG	Human glioma
Vero	African green monkey kidney fibroblasts.
